# TRIM29 upregulation contributes to chemoresistance in triple negative breast cancer via modulating S100P-β-catenin axis

**DOI:** 10.1186/s12964-025-02233-9

**Published:** 2025-05-26

**Authors:** Qitong Wu, Sumit Siddharth, Deepak Verma, Sheetal Parida, Dipali Sharma

**Affiliations:** Department of Oncology, The Sidney Kimmel Comprehensive Cancer Center, Johns Hopkins University School of Medicine, 1650 Orleans Street, CRB 1, Rm 145, Baltimore, MD 21231 USA

## Abstract

Triple negative breast cancer, an inherently aggressive disease, is further impaired by the limited therapeutic options and chemotherapy-resistance; hence, elucidating the signaling nodes underlying chemotherapy resistance is of major interest. Focusing on the differentially expressed genes in recurrent TNBC, we identified TRIM29, a ubiquitin ligase belonging to TRIM family, as a uniquely enriched protein in chemoresistant TNBC. Here, we demonstrate that chemoresistant TNBC cells are inherently aggressive, exhibiting elevated growth and migration potential compared to chemosensitive cells, and in particular, they possess higher TRIM29 expression whose expression level modulation results in altered chemosensitivity. TRIM29 overexpression reduces chemotherapy response whereas TRIM29 knockout not only increases chemosensitivity but also reduces TNBC tumor growth. Tumor-dissociated cells maintain TRIM29 knockout status as well as exhibit similar functional alterations as chemoresistant TNBC cells. Mechanistically, RNA-sequencing of parental-chemosensitive, chemoresistant-inherently overexpressing TRIM29 and chemoresistant-TRIM29 knockout TNBC cells reveals a unique set of genes (S100P, SERPINB3, SERPINB4, CEACAM5, CEACAM6 and CDH6) showing significant upregulation with the acquisition of chemoresistance and downregulation with the TRIM29 knockout. Furthermore, an enrichment of β-catenin pathway in chemoresistant TNBC cells is observed. We uncovered a functional network where S100P, a metastasis inducing secretory factor, bidirectionally interacts with TRIM29, and modulates the expression of SERPINB3, SERPINB4, CEACAM5, CEACAM6 as well as β-catenin pathway genes. Showing the functional importance, S100P inhibitor reduces the growth and mammosphere formation in chemoresistant TNBC. Moreover, combining β-catenin inhibitor with chemotherapy shows synergistic inhibition of chemoresistant TNBC cells. Indeed, higher expression of TRIM29, S100P and β-catenin associates with reduced recurrence free survival. This work proposes TRIM29 as an important node that modulates a unique gene network in chemoresistant TNBC and whose biological impact is mediated by modulation of S100P and β-catenin.

**Supplementary Information:**

The online version contains supplementary material available at 10.1186/s12964-025-02233-9.

## Introduction

Breast cancer is the most prevalent cancer among women [1] and it is also one of the leading cause of cancer-related mortality world-wide. Heterogenous nature of breast cancer is evident in the presence of multiple subtypes that are characterized based on the enrichment or absence of estrogen receptor (ER), progesterone receptor (PR) and/or human epidermal growth factor receptor 2 (HER2) expression [2]. Uniquely lacking ER-PR-HER2, triple negative breast cancer (TNBC) subtype encompasses multiple molecular sub-categories: basal-like 1 (BL1), basal-like 2 (BL2), mesenchymal (M), mesenchymal stem-like (MSL), immunomodulatory (IM), and luminal androgen receptor (LAR) [3, 4]. In many cases, TNBC tumors display multiple molecular characteristics mentioned above hence they cannot be explicitly sub-categorized [5]. Compared with other breast cancer subtypes, the mean prevalence of TNBC is only 12.7% [6, 7], however, TNBC-related mortality represents 40% of all breast cancer-related deaths [8]. TNBC is associated with an increased rate of distant metastasis and relapse [8, 9] which translates to diminished overall and disease-free survival rates [9, 10]. Indeed, the mortality rate within 5 years of diagnosis for TNBC patients is 70%, compared to 44% for all other subtypes [11]. While various endocrine therapies and anti-HER2 therapies +/- chemotherapy regimens can be utilized to combat the Luminal A/B and HER2 + breast cancer subtypes, TNBCs are majorly treated with chemotherapy [12–16]. Unfortunately, a large number of TNBC patients do not respond to chemotherapy [17] and attain drug resistance [18]. Inherent molecular heterogeneity and development of chemoresistance in TNBC contribute to its aggressive tendency to metastasize and relapse [5, 19]. Multiple molecular factors may contribute towards the development of chemoresistance including drug efflux pumps, altered DNA repair, impaired metabolism, enrichment of chemoresistant cancer stem cells (CSCs) as well as altered tumor microenvironment. Among these factors, drug efflux transporters are most directly involved in reducing drug efficacy by simply pumping the chemotherapeutic drugs out of cancer cells in an ATP-dependent manner [20]. Indeed, upregulation of efflux transporters, breast cancer resistance protein (BCRP), multidrug resistant associated protein-1 (MRP1) and P-glycoprotein (P-gp) is evident in breast cancer cells [21]. Of note, TNBCs are more likely to overexpress multi-drug-resistant protein-1 (ABCC1/MRP1), multi-drug-resistant protein–8 (ABCC11/MRP8), and the breast cancer resistance protein (ABCG2/BCRP) compared to other breast cancer subtypes [20, 22, 23], which may contribute to poor response to chemotherapy. Selective enrichment of chemoresistant CSCs is another important phenomenon that contribute considerably to chemoresistant phenotype [24]. CSCs show hyperactivation of oncogenic signaling pathways as well as antiapoptotic pathways which help overcome the effect of drugs [25]. Interestingly, higher expression of multiple CSC-related protein markers, such as ABCG2, ALDH1, EpCAM, CD24, CD44 and CD10 is positively associated with development of chemoresistance [26, 27]. Several alterations in the tumor microenvironment pertaining to cancer-associated fibroblasts, tumor-associated macrophages and extracellular matrix have been shown to contribute to chemoresistance [28]. In addition, several ncRNA, including miRNAs and IncRNAs in conjunction with their mRNA targets participate in the development of chemoresistance [28]. Despite multiple insights, molecular mechanisms underlying chemoresistance in TNBC remain elusive, and chemoresistant TNBC continues to be an important area for research owing to the high clinical relevance.

TRIM29 is a member of the TRIM family of proteins which contain a B-box type 1, a B-box type 2, a RING, and three zinc-binding domains [29–32]. TRIMs participate in cell differentiation, development, apoptosis, and tumorigenesis [33]. Since TRIM29 is involved in Ataxia telangiectasia [34], it is also recognized as ATDC (Ataxia telangiectasia group D complementing). TRIM29 impacts several biological processes, including mediation of inflammatory response, cell signaling, translocation of protein, cell cycle regulation, autophagy, and apoptosis [35, 36]. Notably, TRIM29 is overexpressed in lung cancer [37], osteosarcoma [38], pancreatic cancer, gastric cancer [39] and colorectal cancer [40]. TRIM29 overexpression leads to an increased CD44 expression, which activates the Wnt-β-catenin signaling pathway and results in enhanced migration and invasion in colorectal cancer [40]. Similarly, TRIM29 expression mediates increased viability, migration, and mammosphere via activation of MAPK and β-catenin in cholangiocarcinoma [41]. However, overexpression of TRIM29 decreases proliferation in the Luminal A breast cancer cells [42] and HER2 enriched breast cancer in vitro [43]. Interestingly, the expression levels and biological role of TRIM29 varies among different breast cancer subtypes [43], hence, remain elusive.

While many studies have put forth multiple mechanisms underlying the chemoresistance in TNBC, we aimed to identify some upstream node(s) that may modulate key oncogenic pathways/players and have a significant role in the chemoresistant TNBC. Analysis of differentially expressed genes (DEGs) in recurrent TNBC samples led to the identification of TRIM29 whose higher expression significantly associates with poor overall survival in TNBCs. We observed that chemoresistant TNBC cells exhibit higher growth, migration and mammosphere-formation along with higher expression of TRIM29. Indeed, modulation of TRIM29 impacts chemosensitivity of TNBC cells, and TRIM29 knockout abrogates the expression of several oncogenic candidate proteins including S100P. Our mechanistic studies reveal the presence of TRIM29-S100P-β-catenin network in TNBC chemoresistance.

## Materials and methods

### In silico data analyses

*Differentially Expressed Gene Analysis*: Normalized mRNA data from the Cancer Genome Atlas Breast Cancer (TCGA-BRCA) database was acquired from the Broad GDAC Firehose (https://gdac.broadinstitute.org/) and reads were arranged based on breast cancer subtypes. GSE43502 data [44] were downloaded from Gene Expression Omnibus database (https://www.ncbi.nlm.nih.gov/geo/). Differentially expressed genes (DEGs) analysis was performed using iDEP.93 [45] for TCGA and GSE43502 data set. DEGs were shown in a volcano plot using R tool Enhance Volcano with default settings (GitHub submission). Log fold change and adjusted *p*-values were used to create the plots. Overexpressed genes were compared to the chemo-resistance signature driven from GSE43502. Common genes between GSE43502 (≥ 1.0 FC) and TCGA (≥ 3 FC) were selected for further analysis. *Survival analysis*: Kaplan-Meier plots were generated using the following website: http://kmplot.com/analysis/. *Bee swarm plots and Violin plots*: We utilized the web-based bc-GenExMiner v4.7 (https://bcgenex.ico.unicancer.fr/BC-GEM/GEM-Requete.php?mode=8) tool to analyze the expression level of TRIM29 in available RNAseq datasets of “TNBC(IHC) and/or Basal-like (PAM50)” using default settings (Welch’s test) [46]. Expression of TRIM29 in TNBC patients categorized through IHC status was shown as bee swarm plots. Expression of TRIM29 in TNBC patients categorized through PAM50 status was presented as violin plots. *UALCAN analysis*: Protein expression of TRIM29 in breast cancer were acquired from: http://ualcan.path.uab.edu [47]. *CHAT analysis*: Result of Cancer Hallmark Analysis of TRIM29 were acquired from: https://chat.lionproject.net [48]. *Correlation analysis*: Single sample gene set enrichment analysis (ssGSEA) was performed on Genepattern (Broad Institute web-based tool) [49]. The oncogenic signature “C6” chip was used to analyze raw reads of TNBC and other subtypes from TCGA. Enriched scores and raw reads of each patient were used to perform Pearson correlation, and highly correlated pathways were selected for further analysis and validation. Correlation plots were created through GraphPad Prism 8. A cutoff of 0.2 for 100 samples based on “2/√no of samples” was used to choose positively correlated pathways and genes.

### Cell culture and reagents

Human TNBC cell lines MDA-MB-231, HCC1806 and HCC70 were obtained from American Type Culture Collection (ATCC, Manassas, VA). Early passages of frozen cells were revived from nitrogen vapor stocks. Short tandem repeat testing was performed for authentication. All cells were maintained at 37 °C in 5% CO_2_ with 95% humidity. MDA-MB-231 cells were cultured in DMEM (Corning, Glendale, AZ) supplemented with 10% fetal bovine serum and 1% antibiotic-antimycotic. HCC1806 and HCC70 were maintained in RPMI (Corning, Glendale, AZ) supplemented with 10% fetal bovine serum and 1% antibiotic-antimycotic. *Carboplatin-Resistant Cell lines*: For the development of resistant cell lines, 5 µg/ml carboplatin (Sigma-Aldrich, St Louis, MO) was added to culture medium every two weeks for 48 h. Then, cells were maintained in normal complete culture medium until the next treatment. Resistant cells used in all the experiments have undergone a minimum of 15 cycles of treatment. For immunohistochemistry, immunoprecipitation, immunofluorescence, and western blot, anti-TRIM29 (66 kDa, SC-376125), β-catenin (92 kDa, SC-7963) and SNAI1 (29 kDa, SC-271977) were procured from Santa Cruz Biotechnology (Dallas, TX). Anti-S100P (10 kDa, ab133554) was purchased from Abcam (Cambridge, United Kingdom). Antibodies against vimentin (57 kDa, D21H3) and N-cadherin (140 kDa, 13116) were obtained from Cell Signaling Inc. (Danvers, MA). βActin (42 kDa, A5441) was procured from Sigma-Aldrich (St. Louis, MO).

### CRISPR knockout

To establish carboplatin resistant HCC1806 with genetic knockout of TRIM29, we selected the guiding oligonucleotide as recommended by the CRISPR online design tool (CRISPick). The forward sequence is 5’-GAAGGAGAAGGACCGCATCA-3’, whereas the reverse sequence is 5’- TGATGCGGTCCTTCTCCTTC-3’, targeting the open reading frame of TRIM29 in exon2. After the oligonucleotides were annealed, the guide was ligated into BsmBI- digested lentiCRISPRv2 plasmid (Addgene, Watertown, MA). Subsequently, calcium phosphate was used to co-transfect the annealed plasmid along with the plasmids containing viral components into HEK293T cells. Media with viral particles was obtained from HEK293T after 48 h of incubation. After filtration, virus-containing media was used to transduce target cells (carboplatin resistant-HCC1806). Cells with successful infection were selected in 1 µg/ml puromycin using a single cell selection strategy. Clones successfully surviving the screen were tested for fidelity of knockout via RT-PCR and immunoblotting.

### Cell viability and clonogenicity

*MTT Assay*: Cells were seeded in 96-well plates (4000 cells/well) and incubated overnight. Drugs were added for 48 h before addition of MTT (Sigma-Aldrich, St Louis, MO) following manufacturer’s protocol. The assay estimates the reduction of 3-(4,5-Dimethylthiazol-2-yl)-2,5-Diphenyltetrazolium Bromide). The viability of cells was calculated in Excel and is graphically represented. *Trypan Blue Exclusion Assay*: Cells were seeded in 24-well plates (5000 cells/well) and incubated overnight. Drugs were added for 48 h. Fuchs-Rosenthal Counting Chamber was used to count cells under phase contrast microscopy. Viable cells were counted and represented graphically. *Clonogenic Assay*: Cells were seeded in 12-well plates (1000 cells/well) and incubated overnight. They were treated with drugs for 48 h, and then were replaced with culture media without drug and incubated for 5–8 days. Colonies were fixed with formalin before staining with 0.1% crystal violet.

### Spheroid migration assay, transwell migration assay and scratch migration assay

*Spheroid Migration Assay*: Tumor spheroids were generated by resuspending cells (20000 cells/well) in an 0.5% agarose-coated 96-well plate and cultured on an orbital shaker for 48 h at 37 °C in 5% CO_2_ with 95% humidity. Intact tumor spheroids were selected and plated on 12-well plate with complete culture media with or without drug for 4 days to allow proper attachment and migration. From day 4 to day 10, spheroids were observed and photographed daily. Quantification of spheroid migration was measured by ImageJ software (Fiji). Speed of migration was calculated and plotted using Excel. *Transwell Migration Assay*: Cells were resuspended with serum free media and seeded onto the upper chambers of transwell inserts (BioCoat Control Inserts, Corning, Glendale, AZ). Media supplemented with 10% serum was added in the lower chambers and incubated for 48 h. Post incubation, the non-migrated cells were removed using cotton swabs and the migrated cells were fixed with formalin and stained using crystal violet (0.1% crystal violet). *Scratch Migration Assay*: Cells were seeded in each well of the ibidi Culture-Insert 2 well (ibidi USA, Fitchburg, WI) (70,000 cells/well) in 35 mm cell culture dishes overnight to allow the formation of monolayer. After removal of the Culture-Insert, cell monolayer was washed with PBS, then it was replaced with fresh culture media. Plates were photographed immediately at 0 h, and the migrating cells were followed for various time intervals. Quantification of wound closure was performed by Image J (Fiji) software. Speed of wound closure was calculated and plotted in Excel.

### Solid mammosphere assay

Solid mammosphere formation assay was performed following the protocol mentioned earlier [50]. In brief, 2 × 10^4^ cells were seeded in 2 ml of mammosphere media containing methyl-cellulose, supplemented with 1% penicillin/streptomycin, B27 (1:50) (Life Technologies, Carlsbad, CA), 5 µg/ml insulin, 1 µg/ml hydrocortisone (Sigma-Aldrich, St Louis, MO), 20 ng/ml EGF (R&D Systems, Minneapolis, MN), 20 ng/ml basic fibroblast growth factor in 30 mm ultra-low attachment plates for 7 days. Mammospheres were stained with filtered crystal violet (0.1% crystal violet) for 1–2 h before imaging. Quantification was performed with Image J (Fiji) software.

### RNA isolation and quantitative RT-PCR

Cells were lysed in TRIzol Reagent (Life Technologies Inc., Rockville, MD) followed by RNA isolation using chloroform-isopropanol method, and then cDNA was synthesized via iScript cDNA Synthesis Kit (Bio-Rad Hercules, CA, USA). Go Taq Green Master Mix (Promega) was used to perform RT-PCR. Actin and GAPDH were used as reference genes for RT-PCR and qRT-PCR. Sequences of reverse and forward primers are given in Table 1.

**Table 1:** Table of forward and reverse primer sequences used in RT-PCR and qRT-PCR

Target	Primer	Sequence (5’ to 3’)
TRIM29	Forward	CATCCTGGAGCAGAACTTCC
	Reverse	TGCTCATCAATGCACCAAAT
Actin	Forward	ACCATGGATGATGATATCGC
	Reverse	ACATGGCTGGGGTGTTGAAG
SerpinB3	Forward	CCTGAAGGTAATATTGGCAGCA
	Reverse	CCAGCGAGGCAAAATGAAAAG
SerpinB4	Forward	TTGGCAATGATACGACACTGG
	Reverse	GCCTGTACATCCTCCAGCAA
Ceacam5	Forward	AGGCCAATAACTCAGCCAGT
	Reverse	GGGTTTGGAGTTGTTGCTGG
Ceacam6	Forward	TCAGCCACTGGCCTCAATAG
	Reverse	TCTGGTCCAATCTGCCAGTC
S100P	Forward	GGAGGAAGGTGGGTCTGAAT
	Reverse	CCACGGCATCCTTGTCTTTT
GAPDH	Forward	AAT CCC ATC ACC ATC TTC CA
	Reverse	TGG ACT CCA CGA CGT ACT CA
C-MYC	Forward	TCAAGAGGCGAACACACAAC
	Reverse	GGCCTTTTCATTGTTTTCCA
MMP7	Forward	TGTATGGGGAACTGCTGACA
	Reverse	AGCGTTCATCCTCATCGAAGT
SLUG β-catenin	Forward	TTGAACATTCCTGGCGCATG
	Reverse Forward Reverse	GGCACTTGGAAGGGGTATTGT GCTGATTTGATGGAGTTGGA TCAGCTACTTGTTCTTGAGTGAA

### Transfection, protein isolation and western blotting

Cells were transfected with TRIM29 overexpression plasmid using Lipofectamine 2000 (Thermo Fisher Scientific) following manufacturer’s protocol. TRIM29/ATDC overexpression plasmid was a generous gift from Dr. Simeone Diane [51]. *Transfection for siRNA*: Cells were either transfected with si-S100P or a scramble control (Santa Cruz Biotechnology, Dallas, TX) using Lipofectamine 2000 following manufacturer’s instructions. *Protein Isolation and Western Blotting*: Total protein lysates were prepared using modified RIPA buffer and quantified using Bradford assay. Equal amounts of total protein lysates were resolved on sodium-dodecyl sulfate polyacrylamide gel and transferred onto PVDF membrane followed by immunoblotting using specific antibodies.

### Immunocytochemistry and immunohistochemistry

*Immunocytochemistry*: Cells were plated in 8-well chambers (Nunc, Rochester, NY, USA) (20,000 cells/well) and incubated overnight. Cells were fixed with 4% paraformaldehyde for 30 min followed by permeabilization with 0.1% Triton-X-100. After blocking with 3% BSA in PBS, cells were incubated with primary antibody in 3% BSA overnight followed by an incubation with TRITC- tagged secondary antibody (Invitrogen, 1:500 dilution with blocking buffer) for an hour at room temperature. DAPI (Thermo Fisher Scientific) was used to stain nucleus before mounting. Mounted slides were visualized under Lieca F800 fluorescent microscope. With oil immersion objective, images were captured at 63X magnification with Lieca Elements software. *Immunohistochemistry*: Tumor tissues were fixed in 10% formalin, embedded in paraffin and sectioned. Tissue sections were probed with desired primary antibodies followed by incubation with HRP conjugated secondary antibody, and developed using DAB peroxidase substrate kit (SK-4100, Vector Laboratories, CA). Slides were visualized with a Nikon microscope (Model eclipse Si RS) at 20X magnification. Images were analyzed by Aperio ImageScope Software, Leica. Quantification of micrographs and IHC was done using Leica Aperio ImageScope, Leica Biosystems.

### Orthotopic-xenograft model

All animal experiments involved in this study were approved by the Johns Hopkins Institutional Animal Care and Use Committee. SCID-NOD mice (female, 6–8 weeks old) were procured from SKCCC animal facility and maintained in-house. Exponentially growing TRIM29 knockout carboplatin-resistant HCC1806 cells (TRIM29KO) and vector control (LentiV2) were resuspended in PBS solution and cell count was adjusted to 1 × 10^8^ per ml. Cell suspensions were mixed with equal parts of Matrigel and each immunodeficient mice received 100 µl mixture (5 × 10^6^ cells/implant) in the fourth mammary fat pad on either side for tumor formation (*n* = 5/group). Tumor growth was regularly measured. At the end of experiment, tumors were excised and processed for further analysis.

### Statistical analysis

Experiments were carried out thrice in triplicates. Statistical significance was evaluated by two-tailed student *t* test using *p* < 0.05 as demonstrative for statistical significance. * Signifies a *p* value of < 0.05; ** signifies a *p* value of < 0.01; *** signifies a *p* value of < 0.001.

## Results

### Elevated expression of TRIM29 is observed in TNBC tumors with recurrent disease

**Fig. 1:**
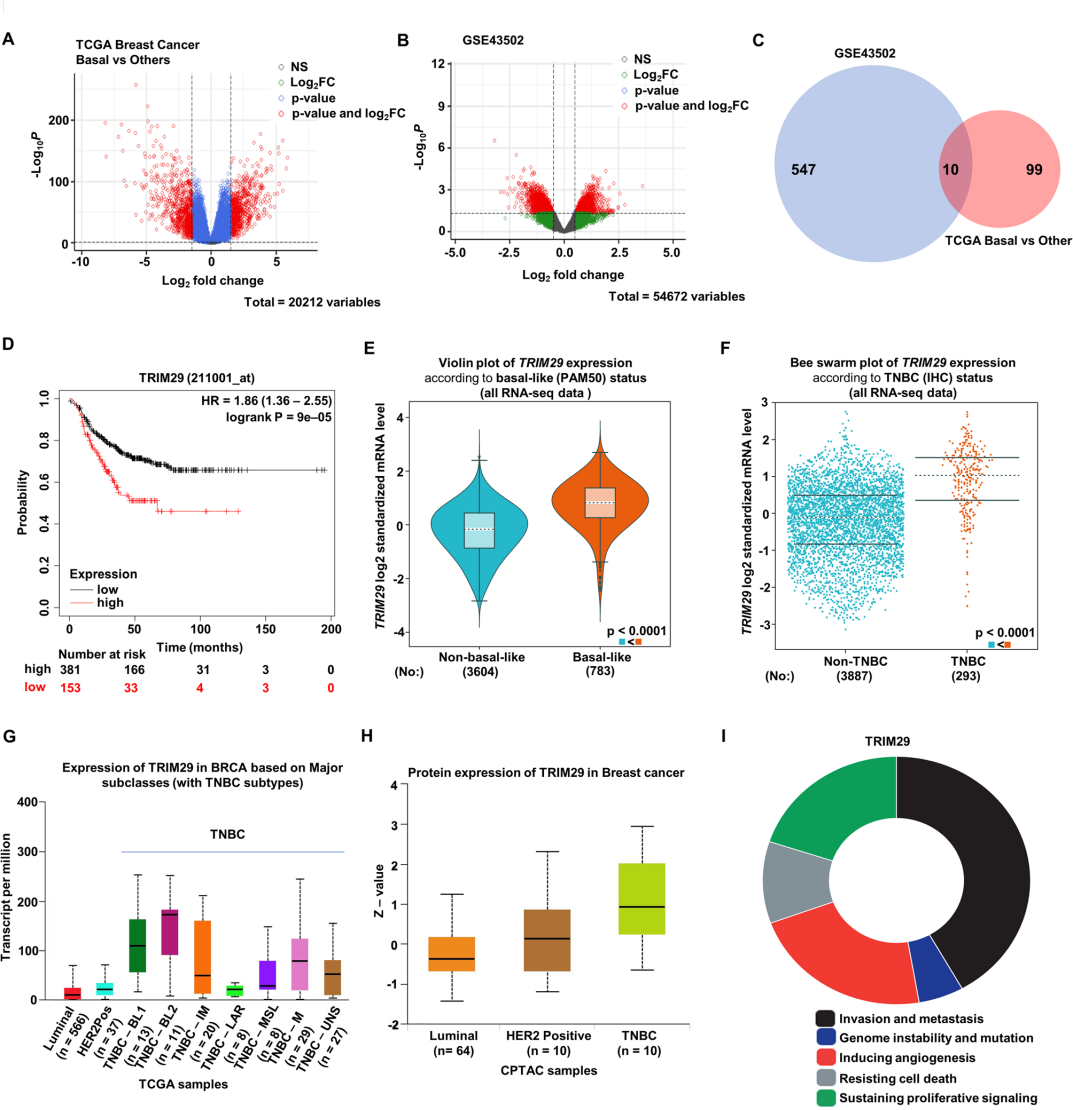
TRIM29 is highly expressed in recurrent TNBC. (**A**) Volcano plot visualizing differentially expressed genes in TNBC tumors compared to luminal and Her2 subtypes. The gene set was acquired from the TCGA database. (**B**) Volcano plot showing differentially expressed genes in TNBC tumors with recurrent disease compared to the ones without recurrence. Gene set used was GSE43502 from Gene Expression Omnibus. (**C**) Venn diagram showing numbers of overlapping genes with LogFC > 1 from TCGA dataset and GSE43502 dataset. (**D**) Kaplan-Meier curves indicating TNBC recurrence-free survival with high- or low- expression of TRIM29. Log-rank tests of survival patterns were used to obtain the *p* values. (**E**) Violin plot representing TRIM29 expression within 4387 breast cancer samples. The breast cancer subtype classification was based on PAM50 status. (**F**) Bee swarm plot representing TRIM29 expression in 4180 breast cancer samples. The breast cancer subtype classification was done via IHC status. (**G**) UALCAN analysis showing TRIM29 expression among different subtypes of breast cancer in TCGA dataset. (**H**) UALCAN analysis showing TRIM29 protein expression level among the three major subtypes of breast cancer from CPTAC dataset. (**I**) Visualization of cancer hallmark distribution of TRIM29 in donut chart. Each color represents a cancer hallmark. The occupied area of each color is proportional to NPMI (normalized point-wise mutual information)

To investigate the functional nodes involved in the chemoresistance of TNBC, firstly, we analyzed the TCGA BRCA dataset and performed a comparative analysis of differentially expressed genes (DEGs) between TNBC and all other subtypes of breast cancer. TNBC samples showed 109 upregulated DEGs (≥ 3 log FC, *p* ≤ 0.05) (Fig. 1A). Owing to our specific interest in genes that are involved in chemoresistance in TNBC, we examined DEGs in TNBC samples among patients with and without recurrent disease (GSE43502, NCBI-GEO). Recurrent TNBC samples showed 557 upregulated DEGs (> 1 log FC, *p* ≤ 0.05) (Fig. 1B). Further analysis was conducted to identify the DEGs that are uniquely enriched in TNBC vs. all other subtypes, and are associated with recurrent TNBC disease. Only 10 DEGs (ACE2, FGFBP1, K6C, KRT16, KRT81, HORMAD1, MMP12, PI3, SHC4, and TRIM29) were found to be overexpressed in recurrent TNBC that were also enriched in TNBC compared to all other breast cancer subtypes (Fig. 1C). To identify functionally important gene(s) that may contribute to a worse prognosis in TNBC, recurrence free survival analyses were performed on the 10 overlapping genes (Supplementary Fig. 1). TRIM29 was found to be associated with worse recurrence free survival (RFS) in TNBC patients with a hazard ratio of 1.86 (*p* = 0.00009) among all other 9 overlapping genes (Fig. 1D, Supplementary Fig. 1, 2). Evaluation of TRIM29 expression pattern among all TNBC patients using RNA-seq data from the TCGA database showed that most TNBC tumors, whether classified via PAM50 (Prediction Analysis of Microarray 50) status (Fig. 1E) or classified based on IHC (immunohistochemistry) status (Fig. 1F), had an elevated expression level of TRIM29 compared to other subtypes of breast cancer. Additionally, data from Clinical Proteomic Tumor Analysis Consortium revealed that most subtypes of TNBC, except for the LAR subtype, exhibited an increased protein expression of TRIM29 compared to Luminal and HER2-enriched breast cancer (Fig. 1G, H). Next, we investigated the role of TRIM29 in cancer via Cancer Hallmark Analysis Tool (Fig. 1I). The visualizations in Fig. 1I were plotted based on normalized pointwise mutual information (npmi), which is an indicator for co-occurrence standard. We observed that TRIM29 contributed to 5 hallmarks of cancer i.e., invasion and metastasis, inducing angiogenesis, sustaining proliferative signaling, resisting cell death, and contributing to genome instability. These results suggested that TRIM29 is not only overexpressed in TNBC but also associates with worse prognosis and recurrent disease.

### Elevated expression of TRIM29 is observed in chemoresistant TNBC cells possessing distinct functional alterations

**Fig. 2:**
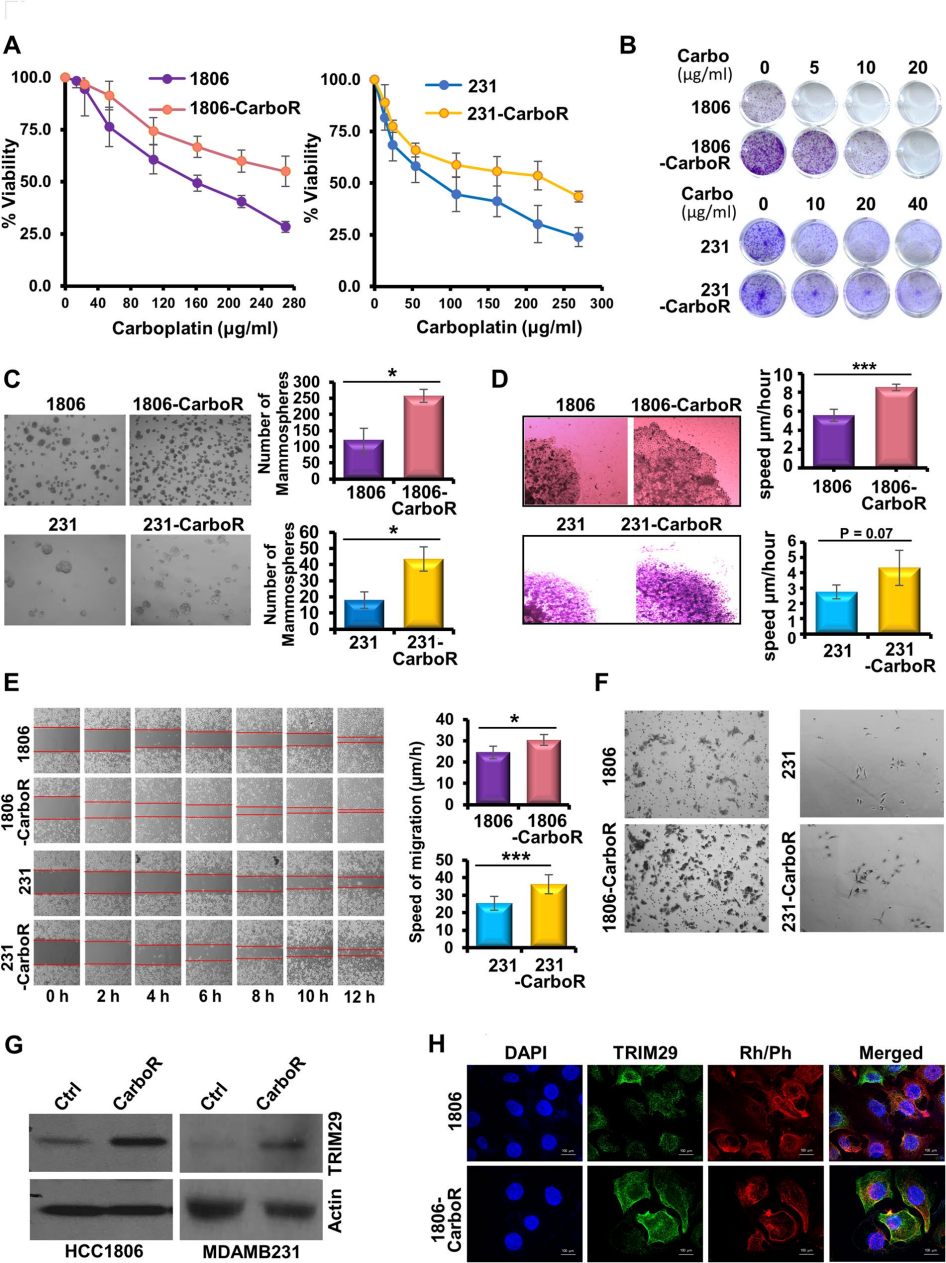
Chemoresistant TNBC cells exhibit distinct functional characteristics and higher TRIM29 expression. (**A**) Result of MTT assay presented as line graph comparing % cell viability between chemoresistant TNBC cells and parental cells upon different concentrations of carboplatin. (**B**) Representative images of colony formation assay of chemoresistant cells and parental cells in different treatment groups. Concentrations of carboplatin were presented in µg per ml. (**C**) Representative images of solid mammospheres formed by chemoresistant TNBC cells and parental cells. Bar graphs show the number of mammospheres. (**D**) Representative images of spheroid migration comparing chemoresistant cells with parental cells. Bar graphs show the speed of migration by the cells from the core. (**E**) Representative images showing the progression of scratch migration assay of parental and chemoresistant cells. Bar graphs indicate the average speed of migration by migrating parental and chemoresistant cells. (**F**) Representative images of transwell migration assay comparing carboplatin-resistant cells with respected parental cells. (**G**) Immunoblotting of TRIM29 in parental and chemoresistant cells. Actin served as the loading control. (**H**) Immunofluorescence analysis of TRIM29 in HCC1806 and 1806-CarboR cells. DAPI was used to stain nuclei. Rhodamine Phalloidin was used to stain F-actin. Scale bar = 25 μm. Data represents *n* = 3 independent experiments. *p ≤ 0.05, ***p ≤ 0.001

To further examine chemoresistance in TNBC, we established chemoresistant TNBC cell lines using carboplatin. HCC1806 carboplatin-resistant cell line (1806-CarboR) and MDA-MB-231 carboplatin-resistant cell line (231-CarboR) were developed by introducing carboplatin treatment in parental cells (1806-Ctrl and 231-Ctrl) for 15 cycles of treatment. Acquisition of carboplatin resistance in TNBC cells was confirmed using MTT assay (Fig. 2A). Both 1806-CarboR and 231-CarboR cells exhibited higher clonogenic potential which remained elevated in response to carboplatin treatment (5–10 µg/ml in 1806-CarboR and 10–40 µg/ml in 231-CarboR cells) (Fig. 2B). Higher growth, proliferation and colony forming ability of the chemoresistant cells compared to parental cells encouraged us to evaluate the tumor-initiating ability of the carboplatin-resistant cells which was assessed via mammospheres assay. As shown in Fig. 2C, both 1806-CarboR and 231-CarboR cells formed a higher number of mammospheres compared to their parental cells suggesting that the carboplatin-resistant (chemoresistant) cells inherently possess a higher tumor-initiating potential compared to TNBC cells that are not resistant to carboplatin. Next, to explore the migration potential of the carboplatin-resistant cell lines compared to parental cells, we performed spheroid-migration and scratch-migration assay. Exhibiting a faster rate of migration, 1806-CarboR cells migrated at a speed of 8.51 μm/hour from the core of the spheroids compared to parental 1806 cells that migrated at a speed of 5.58 μm/hour. Similarly, 231-CarboR cells migrated at a speed of 4.31 μm/hour from the core of the spheroid whereas parental 231 cells migrated at a speed of 2.74 μm/hour (Fig. 2D). Further validation of our observations using a scratch-migration assay showed increased migration potential of CarboR cells. More rapid wound closure within 12 h was observed for chemoresistant TNBC cells in comparison to parental cells with 1806-CarboR and 231-CarboR cells moving at a speed of 30.38 μm/hour and 36.27 μm/hour from the edge of the wound/scratch, respectively, compared to 24.56 μm/hour and 25.38 μm/hour by the parental cells, respectively (Fig. 2E). Likewise, higher number of carboplatin resistant cells (1806-CarboR and 231-CarboR) moved through transwell chambers compared to their corresponding parental cells exhibiting increased chemotactic response (Fig. 2F). Collectively, these results suggested that carboplatin-resistant cells have higher migratory potential than the respective parental cells. Increased growth, clonogenicity and migratory features of chemoresistant cells indicated towards aggressiveness of these cells. Since our in silico studies presented TRIM29 as a potential candidate gene associated with chemoresistance and recurrent TNBC disease, we queried the expression of TRIM29 in our CarboR TNBC cell line models. Indeed, higher expression of TRIM29 was observed in 1806-CarboR and 231-CarboR cells compared to parental 1806 and 231 cells (Fig. 2G). Increased cytoplasmic and membrane bound TRIM29 expression was apparent in 1806-CarboR cells in contrast to 1806 cells (Fig. 2H). These results demonstrated that chemoresistant TNBC cells acquire TRIM29 overexpression corroborating the in silico findings.

### Modulation of TRIM29 expression alters response to chemotherapy

**Fig. 3:**
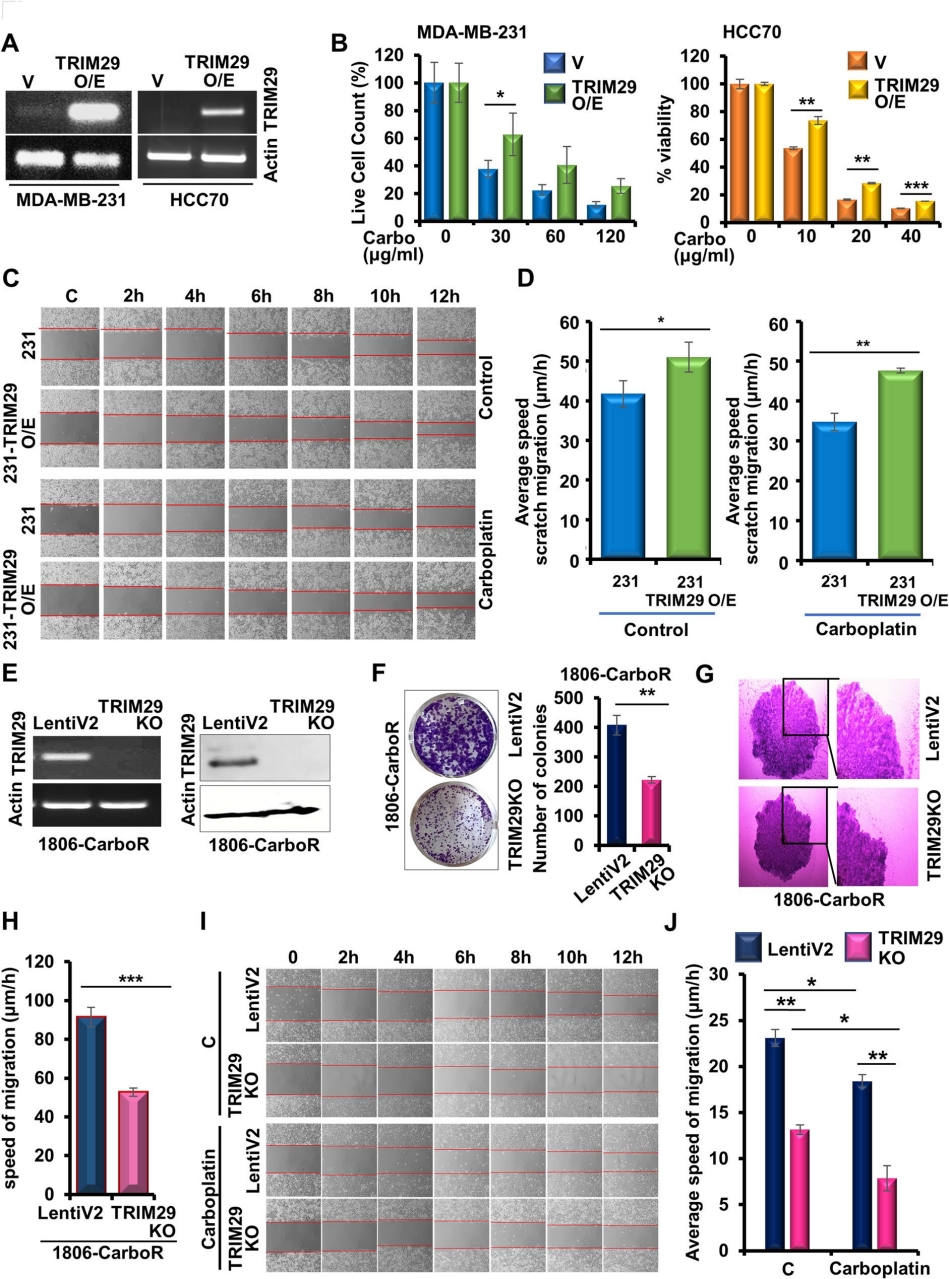
TRIM29 overexpression or knockout modifies the cell viability, migration potential and response to chemotherapy. (**A**) RT-PCR showing TRIM29 expression level in TNBC cells transfected with vehicle or TRIM29-overexpression plasmid. (**B**) Bar graph showing % live cell count in trypan blue exclusion assay and % cell viability in MTT comparing MDA-MB-231 and HCC70 cells treated with transfection vehicle or with TRIM29 overexpression plasmid, in the presence of absence of carboplatin. (**C**) Representative images showing the progression of scratch migration assay of untreated and carboplatin treated MDA-MB-231 cells upon TRIM29 overexpression in comparison to vehicle control cells. (**D**) Bar graphs show the average speed of migration comparing cells with and without overexpression of TRIM29, and carboplatin treatment. (**E**) RT-PCR and immunoblot analysis showing the expression of TRIM29 in HCC1806 carboplatin-resistant cells with TRIM29 knocked out by CRISPR (TRIM29KO) and cells treated with vector (LentiV2). Actin was served as the loading control. (**F**) Representative images of colony formation assay of 1806-CarboR cells upon stable knockout of TRIM29. Bar charts show the numbers of colonies. (**G**) Representative images of spheroid migration comparing LentiV2 and TRIM29KO 1806-CarboR cells. (**H**) Bar graph showing the average speed of spheroid migration comparing LentiV2 and TRIM29KO. Migration distances were recorded for 6 days to calculate the speed of migration. (**I**) Representative images showing the progression of scratch migration assay of LentiV2 and TRIM29KO 1806-CarboR cells in the presence or absence of carboplatin. (**J**) Bar graph showing the average speed of migration comparing LentiV2 and TRIM29KO in the presence or absence of carboplatin. Migration distances were recorded for 6 days to calculate the speed of migration. Data represents *n* = 3 independent experiments. *p ≤ 0.05, **p ≤ 0.01, ***p ≤ 0.001

To address our hypothesis that TRIM29 aids in the acquisition of chemoresistance, we overexpressed TRIM29 in two TNBC cell lines with inherently low TRIM29 expression (Fig. 3A). Trypan blue dye exclusion and cell viability assays indicated that overexpression of TRIM29 in MDA-MB-231 and HCC70 cells imparted resistance to carboplatin (Fig. 3B). Next, we examined the effect of TRIM29 overexpression on the migration ability of TNBC cells using scratch-migration assay. TRIM29-overexpressing MDA-MB-231 cells migrated at an average speed of 51.01 μm/hour compared to control-MDA-MB-231 cells that migrated at an average speed of 41.75 μm/hour. Upon carboplatin treatment, the average speed of TRIM29-overexpressing MDA-MB-231 cells was observed to be 47.64 μm/hour compared to 34.70 μm/hour exhibited by the carboplatin-treated parental cells (Fig. 3C-D). These results indicated that TRIM29 overexpression in TNBC cells confers resistance to carboplatin. Further, as a loss-of-function strategy, we explored whether stable knockout of TRIM29 in carboplatin-resistant TNBC cells enhances their sensitivity towards carboplatin. Towards this, we established 1806-CarboR with a stable genetic knockout of TRIM29 using CRISPR/Cas9 system. The fidelity of stable genetic knockout in TRIM29KO compared to LentiV2 cells was confirmed via RT-PCR and immunoblotting (Fig. 3E). Evaluation of clonogenicity indicated a significant reduction in the colony forming ability of the TRIM29KO-1806-CarboR cells compared to the LentiV2-1806-CarboR cells (Fig. 3F). Interestingly, the migratory potential of carboplatin resistant cells was significantly reduced upon TRIM29 knockout as observed in spheroid migration and scratch migration assays (Fig. 3G-J). In the spheroid migration assay, TRIM29KO-1806-CarboR cells demonstrated a significantly reduced average migration speed of 52.78  μm/hour in comparison to 91.45  μm/hour observed in vehicle control LentiV2-1806-CarboR cells (Fig. 3G, H). Similarly, results from the scratch migration assay indicated a marked decrease in the migratory potential of the TRIM29KO-1806-CarboR cells relative to vehicle control LentiV2-1806-CarboR cells (Fig. 3I). TRIM29KO-1806-CarboR cells displayed an average migration speed of 13.13 μm/hour in the absence of carboplatin, which decreased to 7.84 μm/hour upon carboplatin treatment. Contrastingly, vehicle control LentiV2-1806-CarboR cells migrated at an average speed of 23.08 μm/hour without carboplatin and 18.39 μm/hour with carboplatin (Fig. 3J). Next, we examined whether carboplatin resistant cells show cross resistance to other commonly utilized chemotherapy. Interestingly, we observed that HCC1806-CarboR cells also showed reduced response to doxorubicin and paclitaxel. TRIM29 KO in HCC1806-CarboR cells increased the efficacy of doxorubicin and paclitaxel supporting a wider role of this molecular node in chemoresistance (Supplementary Fig. 3). Collectively, these data suggested that overexpression of TRIM29 induces carboplatin resistance in TNBC cells while knockout of TRIM29 enhances carboplatin sensitivity.

### Loss of TRIM29 reduces tumorigenic potential of chemoresistant TNBC cells

**Fig. 4:**
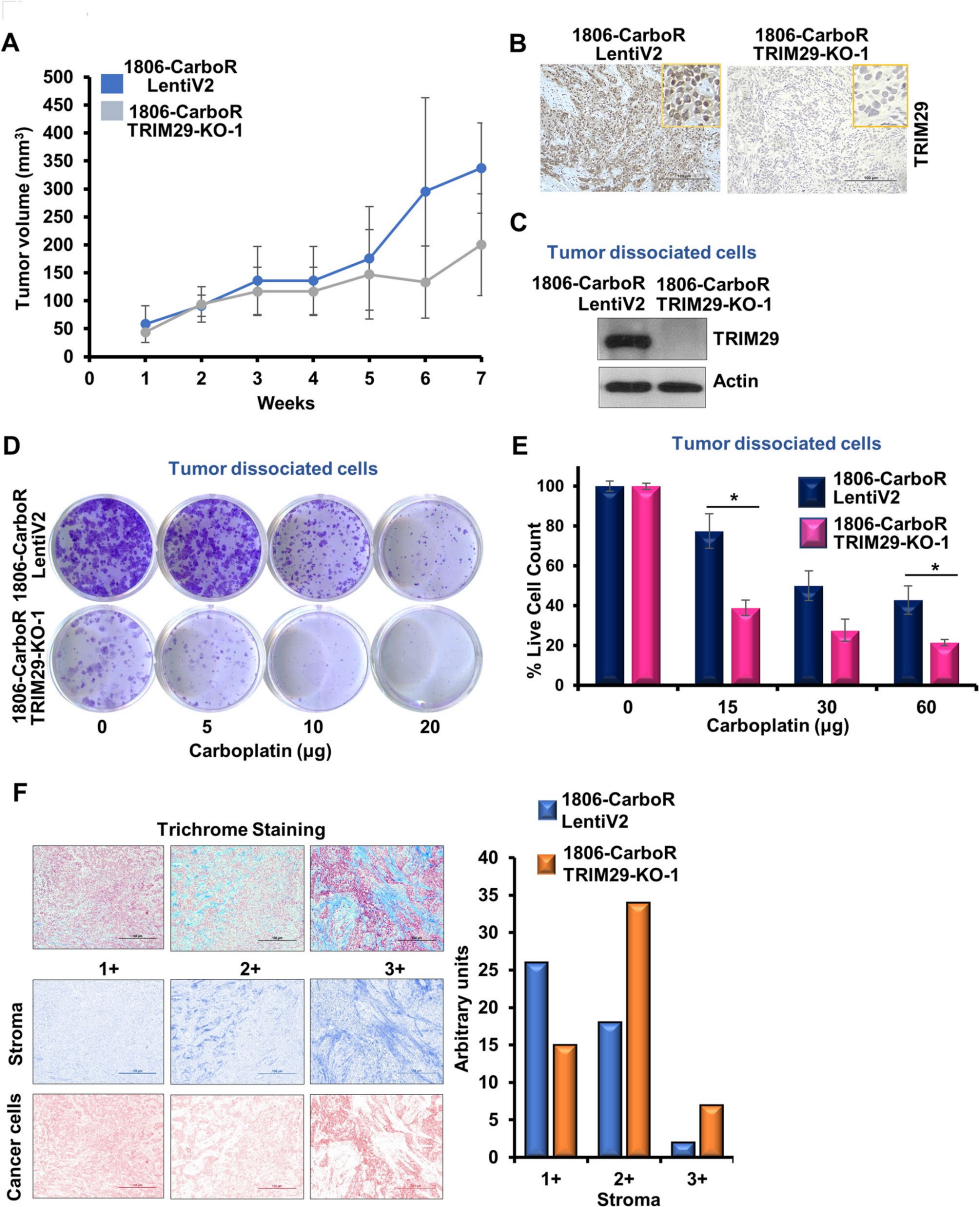
TRIM29 knockout reduces tumor growth of chemoresistant TNBC cells. (**A**) Line graph showing growth of LentiV2 and TRIM29-KO 1806-CarboR cells-derived tumors in NOD/SCID mice. (**B**) Representative IHC images and (**C**) immunoblotting showing TRIM29 in LentiV2 and TRIM29-KO 1806-CarboR cells derived from tumors. Actin was served as the loading control. (**D**) Representative images of colony formation assay of tumor dissociated cells from LentiV2 and TRIM29-KO 1806-CarboR cells-derived tumors, in the presence or absence of carboplatin treatment. (**E**) Bar graph showing % live cell count in trypan blue dye exclusion assay of tumor dissociated cells from LentiV2 and TRIM29-KO 1806-CarboR cells-derived tumors. Data represents *n* = 3 independent experiments. *p ≤ 0.05. (**F**) Representative images of Masson-Trichrome staining showing 1+, 2 + and 3 + staining levels. Tumors were evaluated for the stromal and epithelial content based on the staining intensity and distribution. Bar graph represents the accumulation of stromal cells in 1806-CarboR-LentiV2 and 1806-CarboR-TRIM29-KO- derived tumors (H score)

Encouraged by our in vitro results, we explored the tumorigenic potential of HCC1806-CarboR cells with and without TRIM29 knock-out in immunodeficient mice. Mice harboring tumors derived from TRIM29KO-1806-CarboR cells and LentiV2-1806-CarboR cells were regularly monitored for tumor progression. TRIM29KO-1806-CarboR tumors showed growth reduction in comparison to LentiV2-1806-CarboR tumors (Fig. 4A). As expected, immunohistochemical analysis showed reduced TRIM29 in TRIM29KO-1806-CarboR tumors compared to LentiV2-1806-CarboR tumors (Fig. 4B). Tumor-dissociated cells from TRIM29KO-1806-CarboR tumors showed no TRIM29 expression (Fig. 4C) suggesting that TRIM29 knockout was consistently maintained throughout the tumor development in mice. Ex-vivo assays using tumor dissociated cells further corroborated our in-vitro findings. Clonogenic assay of the ex-vivo model revealed a significant decrease in the colony forming ability of the TRIM29KO-1806-CarboR tumor dissociated cells compared to the LentiV2-1806-CarboR tumor dissociated cells. Also, TRIM29KO-1806-CarboR tumor dissociated cells exhibited increased sensitivity to carboplatin while LentiV2-1806-CarboR tumor dissociated cells maintained their refractory nature towards carboplatin treatment (Fig. 4D). Additionally, cell viability assay using ex-vivo cells demonstrated that TRIM29KO-1806-CarboR tumor dissociated cells were more susceptible to carboplatin treatment than LentiV2-1806-CarboR tumor dissociated cells (Fig. 4E). These findings prompted us to examine the tumors further using Masson-Trichrome staining. Interestingly, we observed that 1806-CarboR-LentiV2-derived tumors possessed lower number of stromal cells while the 1806-CarboR-TRIM29KO-derived tumors had higher accumulation of stromal cells, and consequently a lower number of tumor cells. These results suggest that the higher tumor volume observed in 1806-CarboR-TRIM29KO-derived tumors is contributed by higher prevalence of stromal cells and not by higher number of tumor cells (Fig. 4F). Taken together, these results indicated that loss of TRIM29 reduces the tumorigenic potential of carboplatin-resistant TNBC cells.

### Modulation of TRIM29 in chemoresistant TNBC cells results in distinct molecular changes

**Fig. 5:**
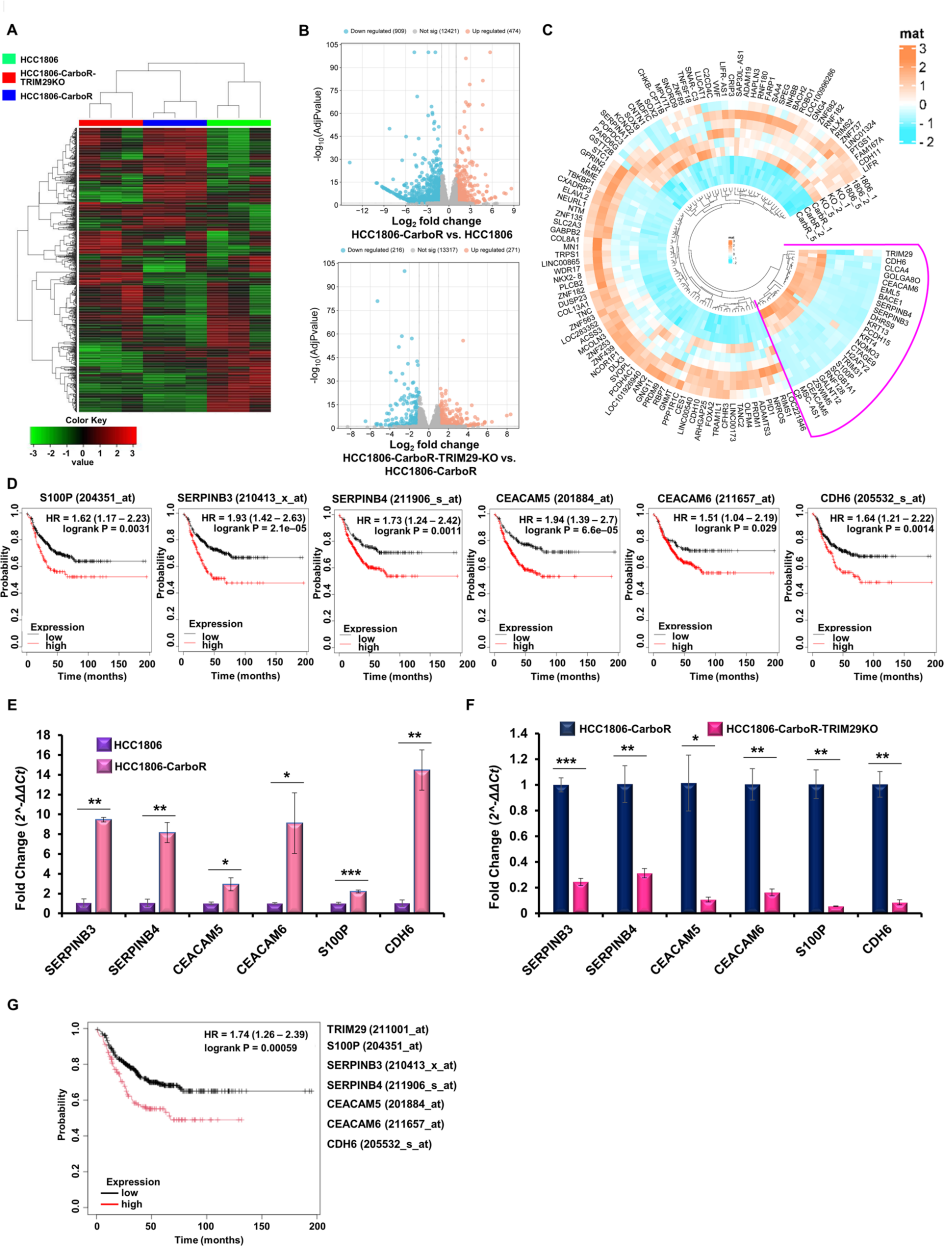
RNA-seq analysis shows that modulation of TRIM29 in chemoresistant TNBC cells alters multiple genes. (**A**) Heatmap representing the differentially expressed genes (DEGs) between HCC1806-parental, HCC1806-CarboR and TRIM29KO 1806-CarboR cells. (**B**) Volcano plots showing the DEGs between HCC1806-CarboR versus HCC1806 cells; and TRIM29KO-1806-CarboR versus HCC1806-CarboR cells, respectively. (**C**) Circular heatmap showing the DEGs specifically downregulated/upregulated in HCC1806-CarboR cells whose expression exhibits opposite trends upon TRIM29 knockout, and similar to HCC1806 cells. Marked up portion represents *n* = 25 DEGs of special interest. (**D**) Kaplan-Meier curves showing the recurrence-free survival (RFS) for the TNBC (using the median of expression as cutoff) with high and low expression of S100P, SERPINB3, SERPINB4, CEACAM5, CEACAM6 and CDH6, respectively. Higher expression of six genes-S100P (Number at risk: low-398, high-136), SERPINB3 (Number at risk: low-360, high-174), SERPINB4 (Number at risk: low-198, high-336), CEACAM5 (Number at risk: low-212, high-322), CEACAM6 (Number at risk: low-136, high-398) and CDH6 (Number at risk: low-359, high-175). High expression of the mentioned genes was associated with worse prognosis. (**E**) Bar graph shows the real-time PCR mRNA expression profile of SERPINB3, SERPINB4, CEACAM5, CEACAM6, S100P and CDH6 in HCC1806 and HCC1806-CarboR cells. (**F**) Bar graph shows the real-time PCR mRNA expression profile of SERPINB3, SERPINB4, CEACAM5, CEACAM6, S100P and CDH6 in HCC1806-CarboR and TRIM29KO-HCC1806-CarboR cells. (**G**) Kaplan–Meier curve showing recurrence-free survival (RFS) for the TNBC patients with high or low mean expression of SERPINB3, SERPINB4, CEACAM5, CEACAM6, S100P, CDH6 and TRIM29. (Number at risk: Low-399, high-135) *p = 0.00059

To further elucidate the molecular mechanisms by which TRIM29 may impart its functional impact on chemoresistant TNBC; HCC1806-parental, LentiV2-1806-CarboR and TRIM29KO-1806-CarboR cells were subjected to RNA-sequencing (RNA-seq) analysis. A differential expression analysis, conducted to characterize the global differences in RNA transcript levels altered with chemoresistance and TRIM29 modulation, exhibited 13,814 differentially expressed genes (DEGs) across three groups. Heatmap showed the unsupervised clustering of DEGs which confirmed separate clusters for HCC1806-parental, LentiV2-1806-CarboR and TRIM29KO-1806-CarboR groups (Fig. 5A). Next, we analyzed the DEGs between LentiV2-1806-CarboR compared to HCC1806-parental and TRIM29KO-1806-CarboR compared to LentiV2-1806-CarboR. A total of 6,416 DEGs (*p* value ≤ 0.05) were found to be differentially upregulated or downregulated among LentiV2-1806-CarboR compared to HCC1806-parental groups while 4,121 DEGs (*p* value ≤ 0.05) were noted to be differentially upregulated or downregulated in TRIM29KO-1806-CarboR compared to LentiV2-1806-CarboR groups (Fig. 5B). While 744 genes showed a differential enrichment or reduction of more than 2-fold (*p* value ≤ 0.05) in resistant cells, only 292 genes exhibited a differential increase/decrease of more than 2-fold (*p* value ≤ 0.05) upon TRIM29 knockout. After further investigations, we observed some distinctly opposite trends in chemoresistant and TRIM29KO-chemoresistant groups. Of note, 98 downregulated genes (2-fold or more downregulation, *p* value ≤ 0.05) in chemoresistant phenotype were upregulated (2-fold or more upregulation, *p* value ≤ 0.05) upon TRIM29 knockout in chemoresistant cells. Interestingly, 25 genes that showed upregulation (2-fold or more upregulation, *p* value ≤ 0.05) with the acquisition of chemoresistance were downregulated (2-fold or more downregulation, *p* value ≤ 0.05) upon TRIM29 knockout. It was also noted that gene expression changes (123 genes presented in a circular heatmap) observed with TRIM29 knockout, for the most part, matched the expression levels in parental chemosensitive cells (Fig. 5C, Supplementary Fig. 4). Further, we questioned the relevance of these 25 differentially enriched/reduced genes in TNBC and performed Kaplan-Meier analysis. Higher expression of six genes-S100P, SERPINB3, SERPINB4, CEACAM5, CEACAM6 and CDH6 out of 25 genes associated with worse prognosis (Fig. 5D). Indeed, higher expression of S100P, SERPINB3, SERPINB4, CEACAM5, CEACAM6 and CDH6 was observed in 1806-CarboR cells compared to 1806-parental cells (Fig. 5E). Notably, TRIM29 knockout significantly decreased the expression level of S100P, SERPINB3, SERPINB4, CEACAM5, CEACAM6 and CDH6 in 1806-CarboR cells indicating that TRIM29 plays a pivotal role in chemoresistant phenotype (Fig. 5F). Subsequently, we interrogated the association of TRIM29 along with S100P, SERPINB3, SERPINB4, CEACAM5, CEACAM6 and CDH6 with recurrence-free survival of TNBC patients and found that higher expression of these seven genes correlated with poor recurrence-free survival outcomes (Fig. 5G). Collectively, these findings underscores the critical role of TRIM29 in chemoresistance in TNBC and uncovers the potential molecular underpinnings.

### Bidirectional interaction between TRIM29 and S100P in chemoresistant TNBC

**Fig. 6:**
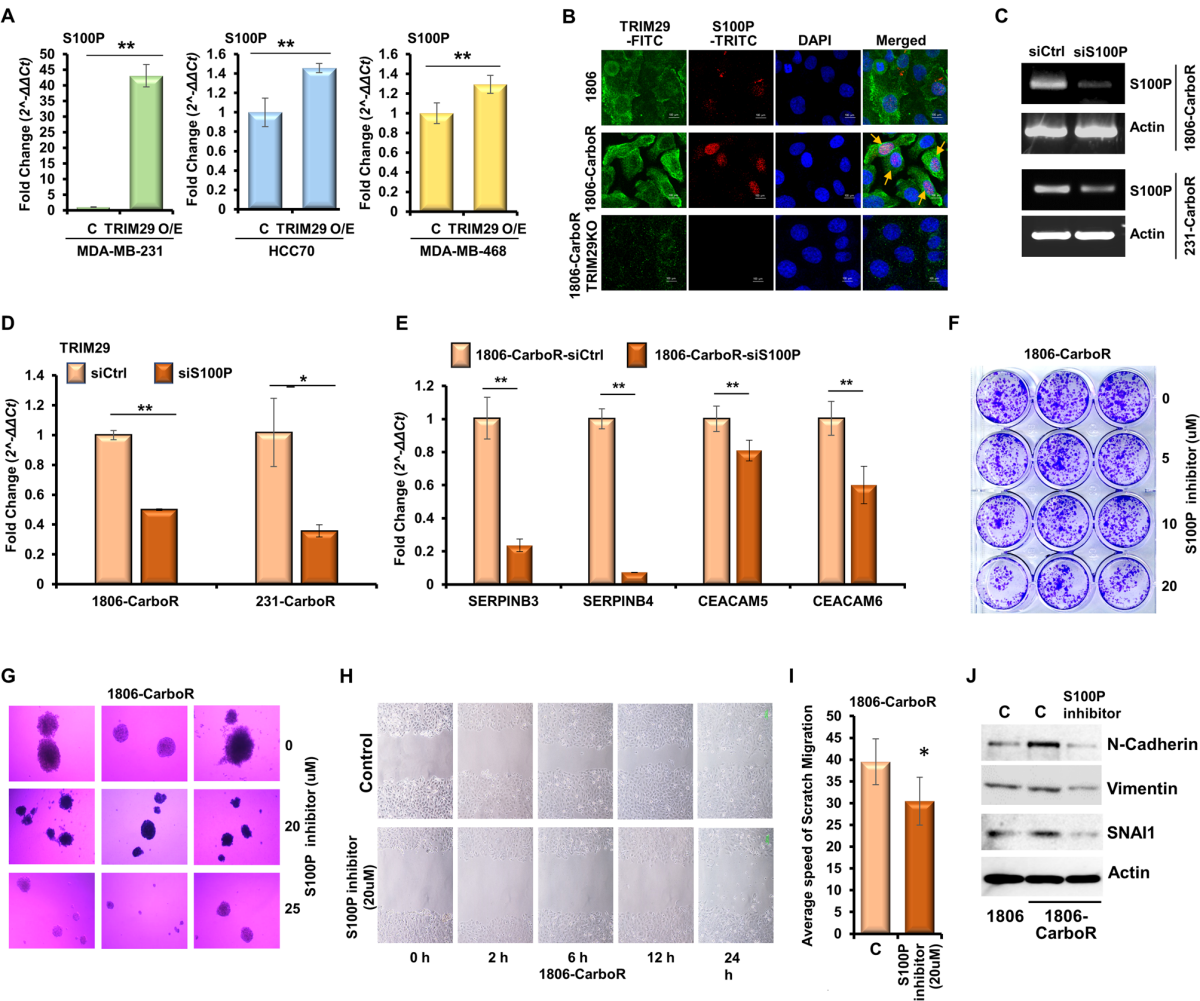
TRIM29 overexpression increases S100P expression while S100P silencing reduces the expression of TRIM29, SERPINB3, SERPINB4, CEACAM5, CEACAM6. (**A**) Bar graph shows the mRNA expression profile of S100P upon TRIM29 overexpression in multiple TNBC cell lines using real time PCR assay. (**B**) Representative images showing the immunocytochemical analysis using S100P and TRIM29 antibodies in HCC1806, HCC1806-CarboR and HCC1806CarboR-TRIM29KO cells. (**C**) Expression level of S100P in carboplatin resistant TNBC cells (1806-CarboR and 231-CarboR) transfected with scramble control or siS100P as indicated. Actin is included as control. (**D**) Bar graph shows the real-time PCR mRNA expression profile of TRIM29 in HCC1806-CarboR and MDA-MB-231-CarboR cells transfected with scramble control or siS100P as indicated. (**E**) Bar graph shows the mRNA expression profile of SERPINB3, SERPINB4, CEACAM5 and CEACAM6 in HCC1806-CarboR cells transfected with scramble control or siS100P as indicated. (**F**) Representative images of colony formation assay of HCC1806-CarboR cells treated with S100P inhibitor. (**G**) Representative images of mammosphere formation assay of HCC1806-CarboR cells treated with S100P inhibitor. **(H**,** I**) Representative images of scratch migration assay in 1806-CarboR cells treated with various concentration of S100P inhibitor as indicated. Bar graph shows the average speed of migration. (**J**) Immunoblot analysis of HCC1806 and HCC1806-CarboR cells treated with 20 µM S100P inhibitor using antibodies as indicated

Thereafter, we focused on S100P, a secretory factor, due to its importance in cancer growth and metastatic progression. S100P is a 95 amino acid member of the S100 family of proteins. Ectopic expression of S100P in pancreatic cancer results in higher tumor growth and metastasis, while the stable silencing results in reduced tumor growth, secondary metastasis and improved sensitivity of cancer cells towards chemotherapy [52, 53]. Immunohistochemical analysis of S100P in 303 breast cancer samples revealed a sevenfold worse prognosis in patients with S100P positive expression compared to patients with negative expression of S100P [54]. Rehbein et al. reported S100P to be upregulated in early as well as advanced stages of lung adenocarcinoma [55]. Firstly, we evaluated the impact of TRIM29 overexpression on S100P expression in TNBC cells, and observed that TRIM29 overexpression led to increased expression of S100P in parental chemosensitive cells (Fig. 6A). Next, we conducted immunocytochemical analysis using S100P and TRIM29 antibodies in HCC1806-CarboR cells. The results showed that S100P and TRIM29 colocalized in HCC1806-CarboR cells while no colocalization was observed in HCC1806 cells or HCC1806-CarboR-TRIM29KO cells (Fig. 6B). We asked if S100P can alter the expression of TRIM29 and related genes (SERPINB3, SERPINB4, CEACAM5, CEACAM6). Since chemoresistant cells inherently overexpress S100P, it was silenced in CarboR cells (Fig. 6C). Silencing of S100P resulted in reduced expression of TRIM29 (Fig. 6D) indicating a bidirectional interaction between S100P and TRIM29. Afterwards, we queried the impact of S100P silencing on the expression of SERPINB3, SERPINB4, CEACAM5, CEACAM6 in chemoresistant TNBC. Of interest, S100P silencing led to reduced expression of SERPINB3, SERPINB4, CEACAM5, CEACAM6 in 1806-CarboR cells (Fig. 6E). Testing the biological impact of S100P inhibition on chemoresistant TNBC cells, we noted a reduction of clonogenic potential (Fig. 6F) and mammosphere formation capability (Fig. 6G) as well as migration potential (Fig. 6H, I) in 1806-CarboR cells upon S100P inhibitor treatment. In addition, elevated expression of N-cadherin, Vimentin and SNAI1 was observed in HCC1806-CarboR cells compared to HCC1806 cells which was reduced upon treatment with S100P inhibitor (Fig. 6J). HCC1806-CarboR-TRIM29KO cells do not respond to S100P inhibitor (Supplementary Fig. 5). Together, these results support an important role of S100P in chemoresistant TNBC.

### Involvement of β-catenin in chemoresistant TNBC along with TRIM29 and S100P

**Fig. 7:**
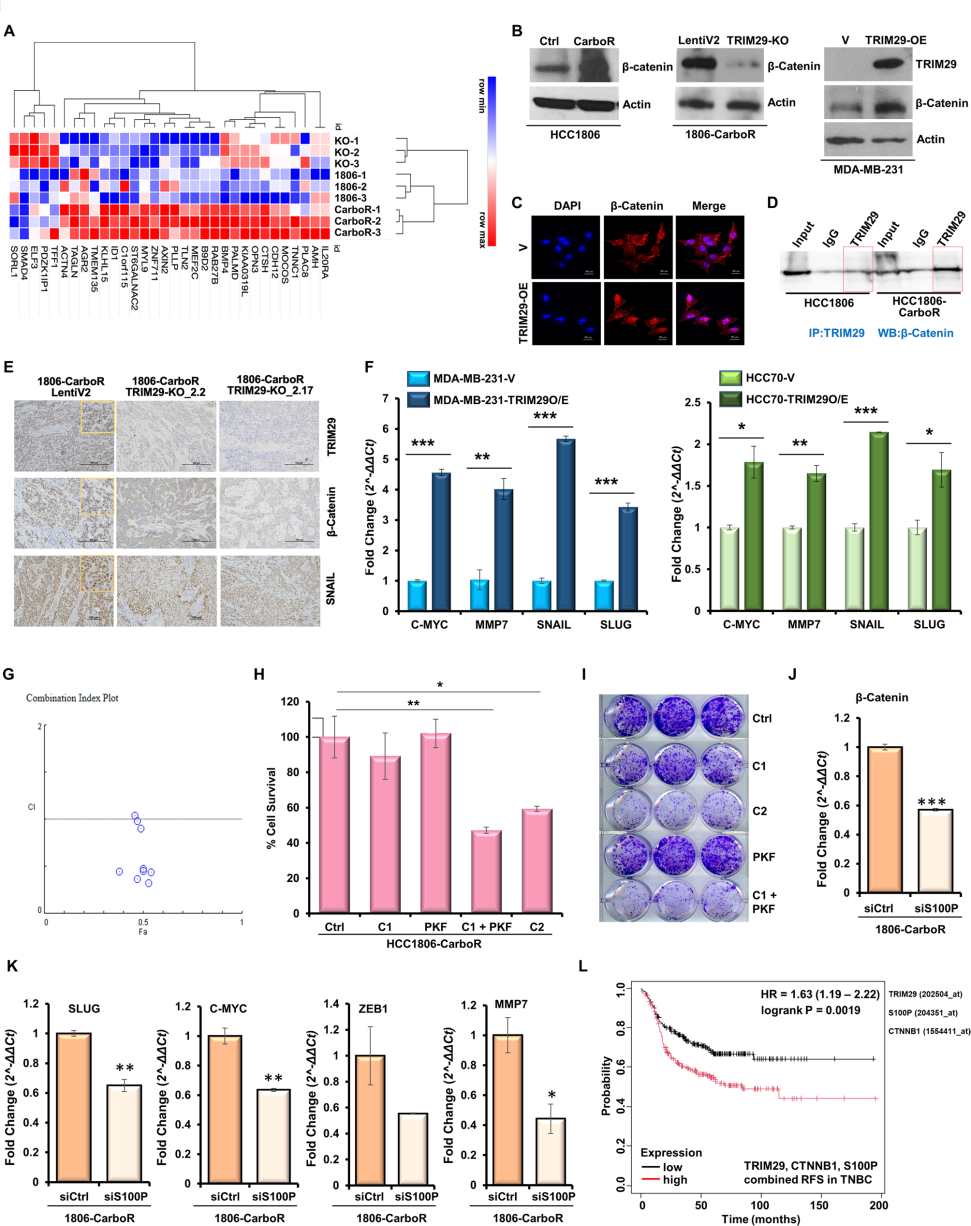
Overexpression of TRIM29 results in increased expression of β-catenin which shows functional significance. (**A**) Heatmap of customized β-catenin pathway signature in HCC1806-parental, HCC1806-CarboR and TRIM29KO 1806-CarboR cells. (**B**) Representative images of immunoblotting of β-catenin in HCC1806-parental and HCC1806-CarboR cells (left panel). Immunoblotting of TRIM29 and β-catenin in LentiV2-1806-CarboR and TRIM29KO-1806-CarboR (center panel). Immunoblotting of TRIM29 and β-catenin in MDA-MB-231 cells transfected with TRIM29 overexpression plasmid (right panel). (**C**) Representative ICC images of β-catenin expression in MDA-MB-231 cells transfected with TRIM29 overexpression plasmid (bottom panel). (**D**) Immunoblot showing β-catenin level immunoprecipitated with TRIM29 in HCC1806 and HCC1806-CarboR cells. (**E**) Representative IHC images of the expression of TRIM29, β-catenin and SNAIL in the tumor sections from LentiV2 and TRIM29-KO 1806-CarboR cells-derived tumors. Scale bar, 100 μm. Inset shows the nuclear staining. (**F**) Bar graph shows the real-time PCR mRNA expression profile of β-catenin-responsive genes (C-MYC, MMP7, SNAIL and SLUG) in MDA-MB-231 and HCC70 cells transfected with TRIM29 overexpression plasmid. (**G**) HCC1806-CarboR cells were treated with various concentration of carboplatin and PKF118-310, alone or in combination. Following the treatment, the cells were subjected to MTT assay and combination index values were calculated using CompuSyn software. CI < 1 shows synergism, CI = 1 shows additivity and CI > 1 shows antagonism (CI = Combination index). (**H**) Bar graph represents % cell survival of HCC1806-CarboR cells treated with the indicated concentration of carboplatin (C, untreated, C1, 5 µg/ml; C2, 10 µg/ml; PKF, 0.3 µM PKF118-310) and PKF118-310 either as monotherapy or in combination, as indicated. (**I**) Representative images of colony formation assay in HCC1806-CarboR cells treated with carboplatin and PKF118-310 either as monotherapy or in combination, as indicated. **(J**,** K**) Bar graph shows the real-time PCR mRNA expression profile of β-catenin (**J**), and β-catenin-responsive genes (SLUG, C-MYC, ZEB1 and MMP7) (**K**) in 1806-CarboR cells treated with scramble or S100PsiRNA, as indicated. (**L**) Kaplan–Meier curve showing recurrence-free survival (RFS) for the TNBC patients (classified with PAM50) with high or low mean expression of TRIM29, CTNNB1 (β-catenin), S100P; (number at risk: low-221, high-221). *p = 0.045. Data represents *n* = 3 independent experiments. *p ≤ 0.05, **p ≤ 0.01, ***p ≤ 0.001

To further uncover the potential oncogenic pathways correlated with TRIM29, we performed a correlation analysis of our RNA-sequencing data using ssGSEA with the C6 oncogenic signature gene-set. This analysis revealed six oncogenic pathways significantly correlated (Pearson correlation ≥ 0.81; *p* value ≥ 0.05) with TRIM29 expression, with two of these pathways (LEF1_UP.V1_DN and BCAT_BILD_EA_AL_UP) specifically corresponding to β-catenin signaling (Supplementary Fig. 6). Further DEG analysis of the curated 32-gene signature for β-catenin signaling in our RNA-seq data clearly showed that most β-catenin signature genes were enriched in 1806-CarboR cells compared to 1806-parental cells while TRIM29KO-1806-CarboR cells showed reduced expression like the parental cells (Fig. 7A). Subsequently, we evaluated the expression level of β-catenin (the key member of β-catenin signaling) in the chemoresistant cells in the presence and absence of TRIM29. Our immunoblotting data indicated that β-catenin was upregulated in HCC1806-CarboR cells compared to parental cells; and this upregulation was reversed upon TRIM29 knockout. Consistent with this finding, overexpression of TRIM29 led to increased β-catenin expression in TNBC cells (Fig. 7B). ICC analysis also corroborated that TRIM29 overexpressing TNBC cells exhibited higher nuclear accumulation of β-catenin (Fig. 7C). We examined the interaction between TRIM29 and β-catenin in HCC1806-CarboR cells which showed that β-catenin immunoprecipitated with TRIM29 at a higher level as compared to HCC1806 cells (Fig. 7D). Further, immunohistochemical analyses of TRIM29KO-1806-CarboR and LentiV2-1806-CarboR cells-derived tumors showed a higher expression of TRIM29, β-catenin and SNAIL (a β-catenin responsive gene) in 1806-CarboR tumors. This was accompanied by decreased expression of β-catenin and SNAIL in the TRIM29KO-1806-CarboR-derived tumors (Fig. 7E). We further focused on the β-catenin target genes (C-MYC, MMP7, SNAIL and SLUG) and observed that their expression increased upon TRIM29 overexpression in TNBC cells (Fig. 7F). Current clinical evidence highlights the limitations of single-agent therapies in TNBC, emphasizing the need for combination therapies. Next, we aimed to explore whether inhibiting β-catenin in combination with carboplatin be sufficient to sensitize the carboplatin-resistant cells. We observed that the combination treatment of PKF + C1 (low-dose-carboplatin) not only outperformed C2 (high-dose-carboplatin) alone, but also acted synergistically (Supplementary Fig. 7, Fig. 7G-H). Thereafter, we examined the colony forming ability of 1806-CarboR cells upon combination treatment (PKF + C1) and compared it with individual monotherapies (C1 or C2). The extent of clonogenicity inhibition attained by low-dose-carboplatin in combination with non-toxic dose of PKF was greater than the high-dose-carboplatin alone (Fig. 7I). As our RNA-sequencing data showed that TRIM29 impacts β-catenin signaling as well as modulates S100P expression, we explored the effect of S100P inhibition on β-catenin and β-catenin-responsive genes’ expression. To our surprise, S100P silencing resulted in reduced expression of β-catenin (Fig. 7J) as well as β-catenin target genes-SLUG, C-MYC, ZEB1 and MMP7 (Fig. 7K) in 1806-CarboR cells. Subsequently, we assessed the association of TRIM29, S100P and β-catenin expression with recurrence-free survival of TNBC patients, and found that higher expression of these three genes correlated with poor recurrence-free survival outcomes (Fig. 7L). TRIM29 (Fig. 1D), S100P (Fig. 5D), and β-catenin (Supplementary Fig. 8) also predict patient outcomes independently. Collectively, these findings support a network between TRIM29, S100P and β-catenin where TRIM29 and S100P can impact the expression of each other in a bidirectional manner, and both TRIM29 and S100P can influence the expression of β-catenin and β-catenin-responsive genes.

## Discussion

TNBC is not only the most aggressive subtype of breast cancer, but is also marred by the lack of targeted therapeutic options owing to the absence of hormone receptor expression and amplification of HER2. TNBCs are also more prone to the development of chemoresistance and metastatic disease, which are the main obstacles to reducing TNBC-related mortality. Here, we identified TRIM29, an important member of TRIM family, as a uniquely enriched protein in recurrent TNBC (post neo-adjuvant chemotherapy) using in silico data analysis comparing TNBC with recurrence to those without recurrence, as well as to all other subtypes of breast cancer. TRIM29 overexpression at mRNA and protein level is apparent in TNBC compared to all other breast cancer subtypes in TCGA and CPTAC datasets, and its higher expression correlates to worse recurrence free survival in TNBC patients indicating an important role in TNBC. Although TRIM29 is primarily an E3 ubiquitin ligase, our in silico findings indicate that TRIM29 contributes to multiple hallmarks of cancer pertaining to tumor progression and chemo-resistance. These observations prompted us to focus on the role of TRIM29 in chemoresistant TNBC. Directly comparing chemosensitive parental cells to chemoresistant TNBC cells, we noted that chemoresistant TNBC cells inherently harbor an increased growth, clonogenic, mammosphere-forming potential along with a highly migratory phenotype. Particularly significant is that chemoresistant TNBC cells possess higher TRIM29 expression whose expression level modulation results in altered chemosensitivity. Reduced colony forming ability and enhanced carboplatin-sensitivity upon TRIM29 knockout in chemoresistant cells highlights the potential of TRIM29 as a node of therapeutic interest. TRIM29 knockout reduces TNBC tumor growth and tumor-dissociated cells maintain TRIM29 knockout status as well as exhibit similar functional alterations as chemoresistant TNBC cells. These results are supported by studies showing the oncogenic role of TRIM29 in different types of cancers. In non-small cell lung cancer, TRIM29 contributes to oncogenesis by modulation of cell cycle-related proteins [56]. Moreover, TRIM29 has also been shown to contribute to chemoresistance in lung cancer [57] and ovarian cancer [58]. Direct impact of TRIM29 is noticeable in bladder cancer, where Bcl family protein and cyclin D1/E levels are enhanced by TRIM29 through the PKC-NF-κB signaling pathway thus resulting in reduced apoptosis with an increase in proliferation signal [59]. Moreover, by activating DNA methyltransferase 3 A, TRIM29 indirectly suppresses PTEN via epigenetic regulation and contributes to poor prognosis in bladder cancer [60–62]. TRIM29 is also a prognostic factor in cervical cancer [63]. TRIM29 essentially participate in several biological functions pertaining to cancer growth and metastatic progression in several cancer types.

In contrast to multiple other cancer types where TRIM29 is known to function as an oncogene, it has been reported to act as a tumor suppressor in breast cancer in two previous studies. An inverse relationship between TWIST and TRIM29 has been presented with TWIST transcriptionally repressing TRIM29 leading to increased motility and invasiveness of breast cancer cells [43]. A follow up study from the same research group further showed that TRIM29 is induced in response to hypoxia, and it blocks hypoxia-induced TWIST expression [64]. It is interesting to note that TRIM29 expression is altered in multiple breast cancer subtypes and that may explain its heterogenous functional role among different breast cancer subtypes. TRIM29 is differentially methylated in breast cancer subtypes, and its methylation levels correlate well with the expression levels. While reduced expression of TRIM29 is noted in estrogen receptor positive luminal subtypes, robust expression is observed in estrogen receptor negative subtypes especially basal like TNBC group that mirrored normal breast tissue. Interestingly, an inverse correlation is detected with respect to methylation status where TRIM29 is hypermethylated in luminal A and B subtypes however TNBC basal like subtype present hypomethylation explaining higher TRIM29 expression in TNBC [65]. Our study reports a higher expression of TRIM29 in chemoresistant TNBC whose functional importance is tested using multiple assays.

Furthermore, our results advance the understanding of the molecular mechanisms underlying TRIM29 function in chemoresistant TNBC. RNA-seq analyses of parental-chemosensitive, chemoresistant-inherently overexpressing TRIM29 and chemoresistant-TRIM29 knockout TNBC cells show unique trends associated with TRIM29 and chemoresistance. A unique set of 25 genes is identified that is significantly upregulated with the acquisition of chemoresistance and inhibited with the TRIM29 knockout. Interestingly, higher expression of only 6 genes (S100P, SERPINB3, SERPINB4, CEACAM5, CEACAM6 and CDH6) out of these 25 genes shows a clear association with worse recurrence free survival in TNBC indicating a functional network. Also, cumulative higher expression of TRIM29 along with S100P, SERPINB3, SERPINB4, CEACAM5, CEACAM6 and CDH6 associates with poor recurrence-free survival of TNBC patients further strengthening our observation. SERPINB3/SCCA1 has been shown to promote epithelial to mesenchymal transition in mammary epithelial cells [66], and is associated with poor pathological response and poor survival in breast cancer patients treated with chemotherapy [67]. SERPINB4 is recently found to be associated with poor overall survival in TNBC patients [68]. Interestingly, CEACAM5 and CEACAM6 facilitates metastatic progression of breast cancer [69–72], and CDH family is also known for their oncogenic role in several cancers including breast cancer [73]. S100P, widely known for its pro-cancer role across multiple cancer types, has also shown clinical relevance in triple negative breast cancer as its higher expression correlates with recurrence related events and higher metastasis in a cohort of 98 patients [74–76]. Of note, S100P is a metastasis-inducing secretory factor [77] that can influence a wide array of functions and signaling pathways, and its plasma levels show robust association with metastatic breast cancer [78]. We uncovered another interesting aspect of S100P as it bidirectionally interacts with TRIM29, and S100P inhibitor not only inhibits the expression of SERPINB3, SERPINB4, CEACAM5 and CEACAM6 but also inhibits growth and mammosphere formation in chemoresistant TNBC. These studies together present a new molecular axis in TNBC. Another interesting observation of our study is the enrichment of β-catenin pathway in chemoresistant TNBC cells which is modulated by TRIM29 as well as S100P. TRIM29 has been known to associate with β-catenin pathway in few other cancers. TRIM29 activates Wnt/β-catenin pathway contributing to EMT, cell proliferation, colony formation, and migration in cervical cancer. S100P also seems to regulate the activity of β-catenin in endometrial cancer [79, 80]. It enhances the Warburg effect by altering the PKM1/PKM2 ratio strengthening the malignant behavior of colorectal cancer [81]. Combining β-catenin inhibition with chemotherapy exhibits synergistic effect in TNBC growth inhibition thus our findings emphasize a complex interplay between TRIM29, β-catenin and S100P in chemoresistant TNBC.

Our study is a first step to show the involvement of SERPINB3, SERPINB4, CEACAM5, CEACAM6, and CDH6 in chemoresistant TNBC. Indeed, previous studies have shown the importance of these selected genes in breast cancer growth and metastatic progression though few genes are very understudied to date with respect to their specific role in triple negative breast cancer and chemotherapy resistance. Most importantly, we show the central role of TRIM29 in chemoresistant TNBC whose biological effects are mediated via modulating S100P and β-catenin. Our study puts together a gene signature that can be further tested for its clinical relevance especially in chemotherapy resistant TNBC.

## Electronic supplementary material

Below is the link to the electronic supplementary material.

Supplementary Material 1

Supplementary Material 2

Supplementary Material 3

Supplementary Material 4

Supplementary Material 5

Supplementary Material 6

Supplementary Material 7

Supplementary Material 8

## Data Availability

Sequencing data will be provided upon reasonable request.
