# Supplementary material for: TRIM29 upregulation contributes to chemoresistance in triple negative breast cancer via modulating S100P-β-catenin axis
Source: Cell Commun Signal. 2025 May 26;23:244. doi: 10.1186/s12964-025-02233-9 (PMC12107940; doi:10.1186/s12964-025-02233-9)
Supplement: Supplementary file 1 — Supplementary Material 1 [file 12964_2025_2233_MOESM1_ESM.docx]

**
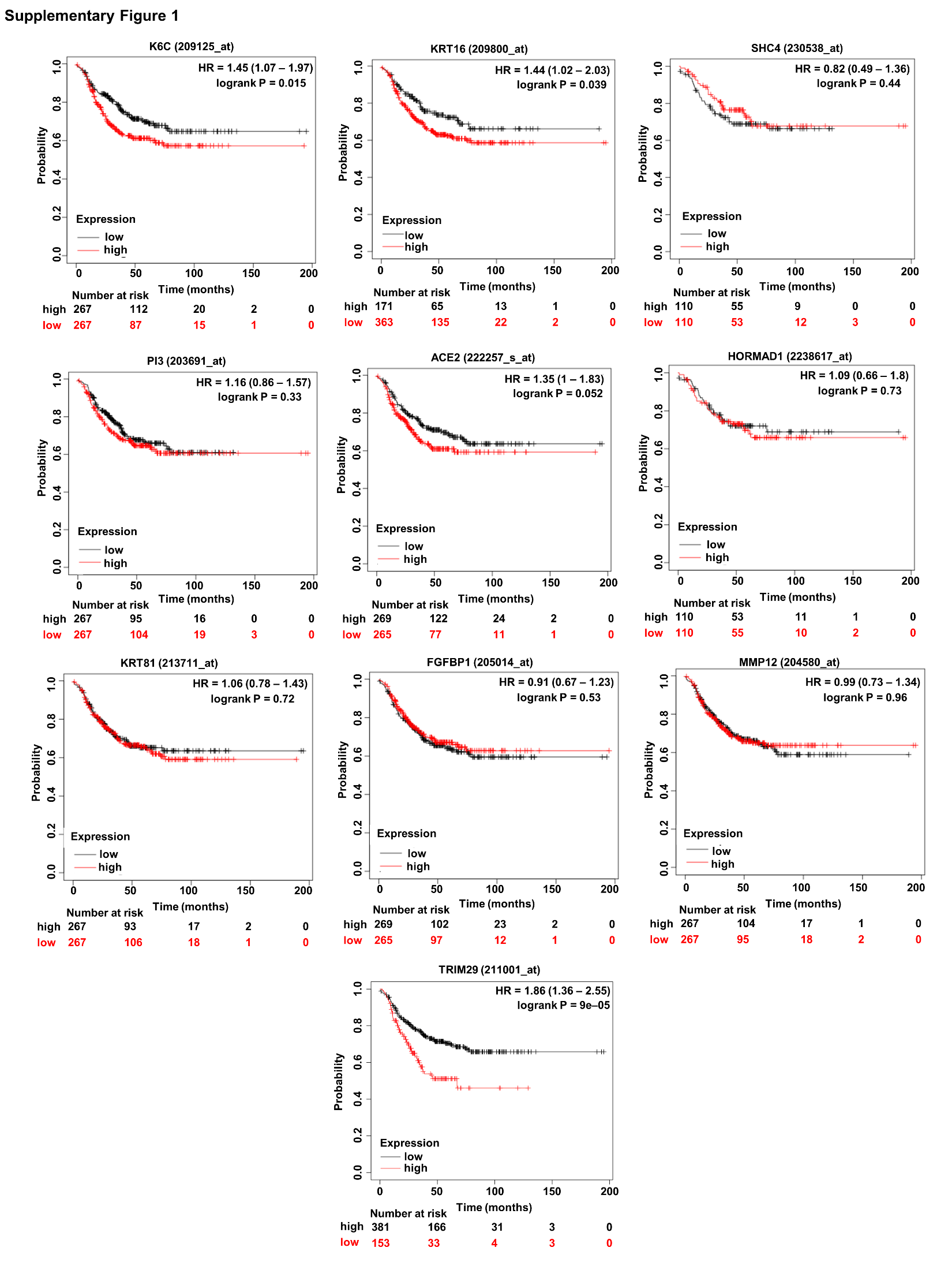
**

**Supplementary Figure 1.** **10 genes were found to be significantly overexpressed among TNBC patients with recurrent disease.** Kaplan-Meier curves indicating TNBC recurrence-free survival with high- or low- expression of overlapping genes in 1C, respectively. Log-rank tests of survival patterns were used to obtain the p values.
