# Supplementary material for: TRIM29 upregulation contributes to chemoresistance in triple negative breast cancer via modulating S100P-β-catenin axis
Source: Cell Commun Signal. 2025 May 26;23:244. doi: 10.1186/s12964-025-02233-9 (PMC12107940; doi:10.1186/s12964-025-02233-9)
Supplement: Supplementary file 2 — Supplementary Material 2 [file 12964_2025_2233_MOESM2_ESM.docx]

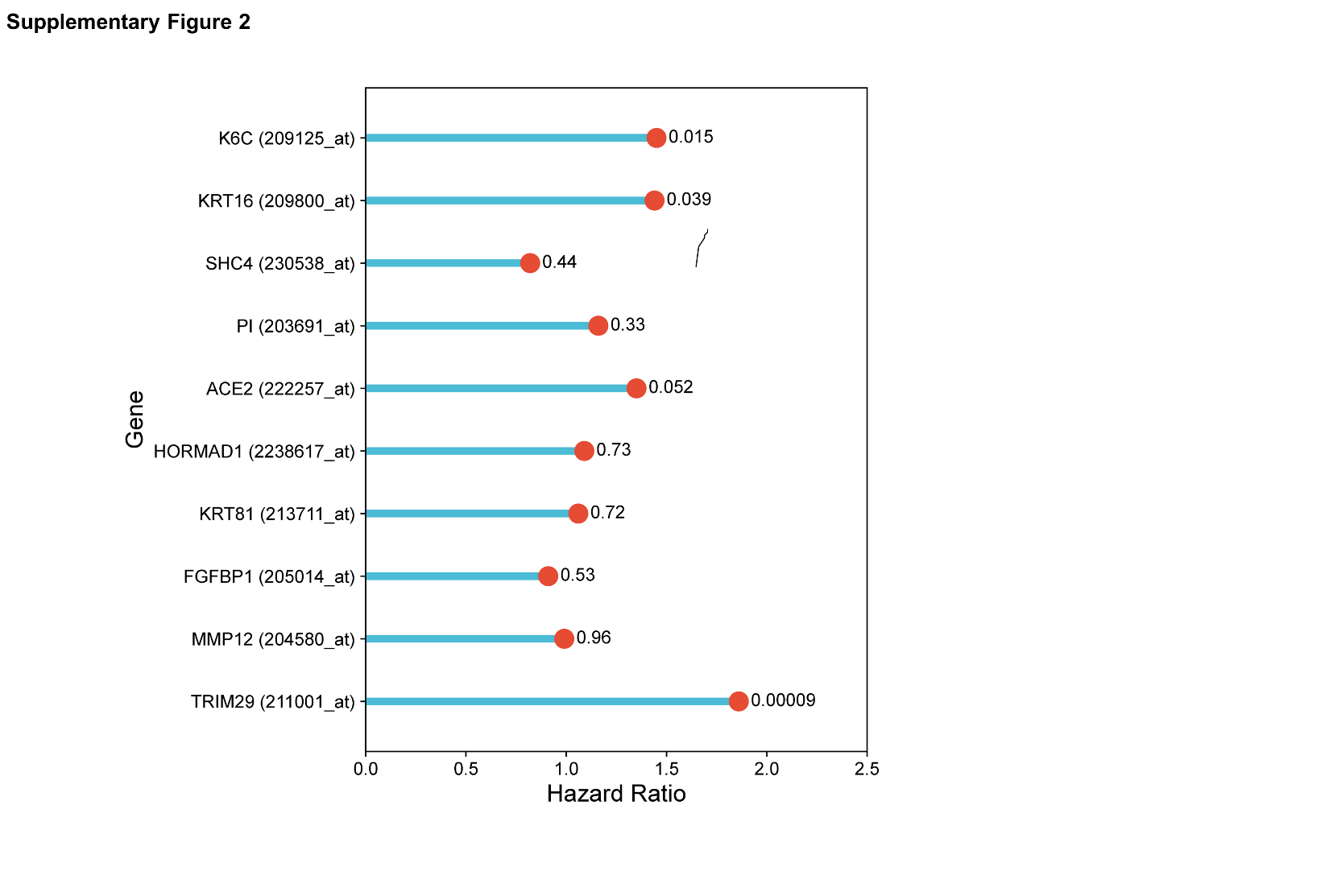


**Supplementary Figure 2. TRIM29 shows superior statistics compared to other 9 selected genes.** The plot shows the hazard ratio along with p-values of overexpressed genes in TNBC patients. TRIM29 is showing a higher significant HR ratio in comparison to the other 9 genes.
