# Supplementary material for: TRIM29 upregulation contributes to chemoresistance in triple negative breast cancer via modulating S100P-β-catenin axis
Source: Cell Commun Signal. 2025 May 26;23:244. doi: 10.1186/s12964-025-02233-9 (PMC12107940; doi:10.1186/s12964-025-02233-9)
Supplement: Supplementary file 3 — Supplementary Material 3 [file 12964_2025_2233_MOESM3_ESM.docx]

**
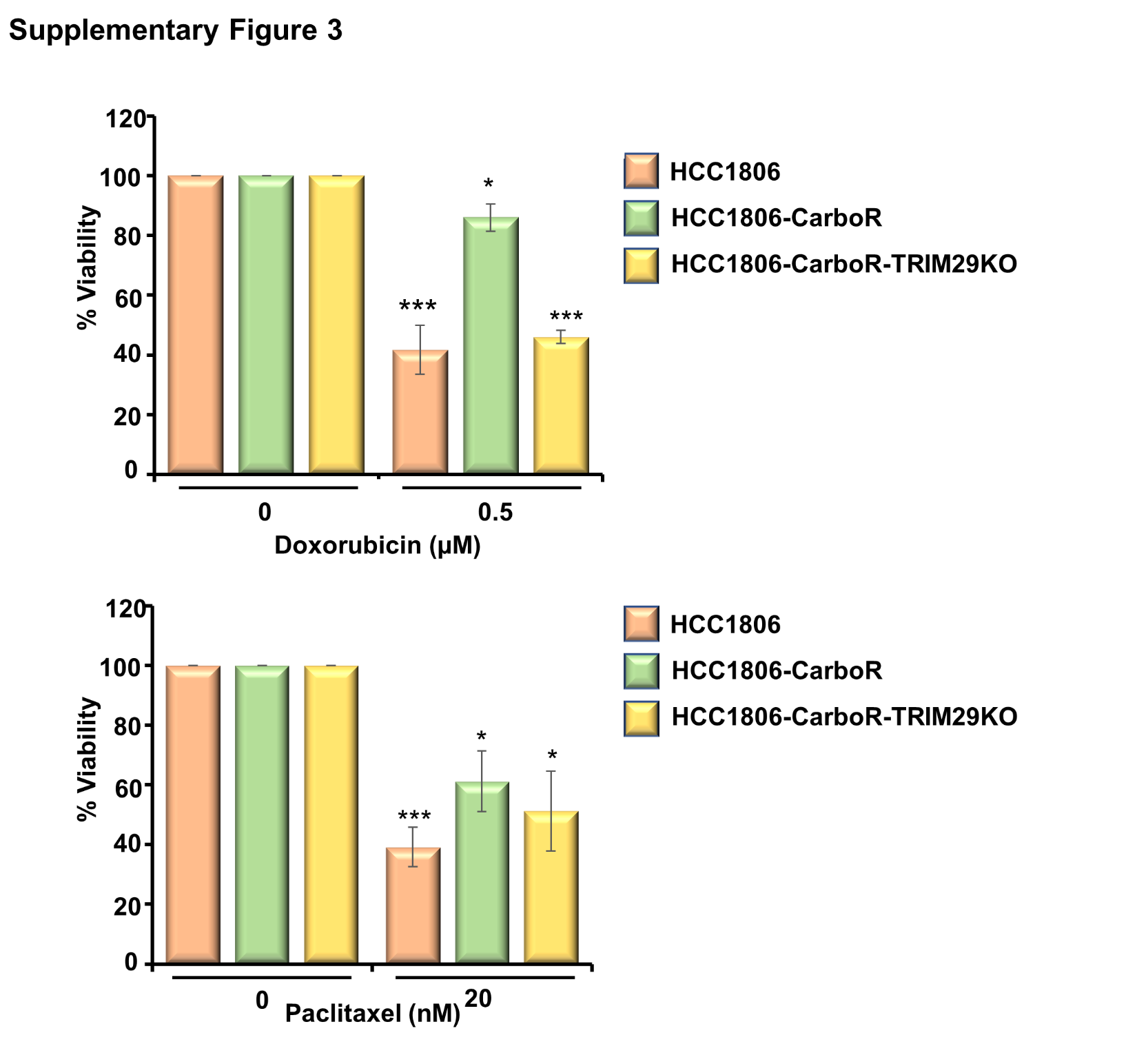
**

**Supplementary Figure 3. Carboplatin-resistance cells are also resistant to additional drugs.** Bar graph showing viability of HCC1806, HCC1806-CarboR and HCC1806-CarboR-TRIM29KO cells treated with doxorubicin or paclitaxel as indicated.
