# Supplementary material for: TRIM29 upregulation contributes to chemoresistance in triple negative breast cancer via modulating S100P-β-catenin axis
Source: Cell Commun Signal. 2025 May 26;23:244. doi: 10.1186/s12964-025-02233-9 (PMC12107940; doi:10.1186/s12964-025-02233-9)
Supplement: Supplementary file 4 — Supplementary Material 4 [file 12964_2025_2233_MOESM4_ESM.docx]

**
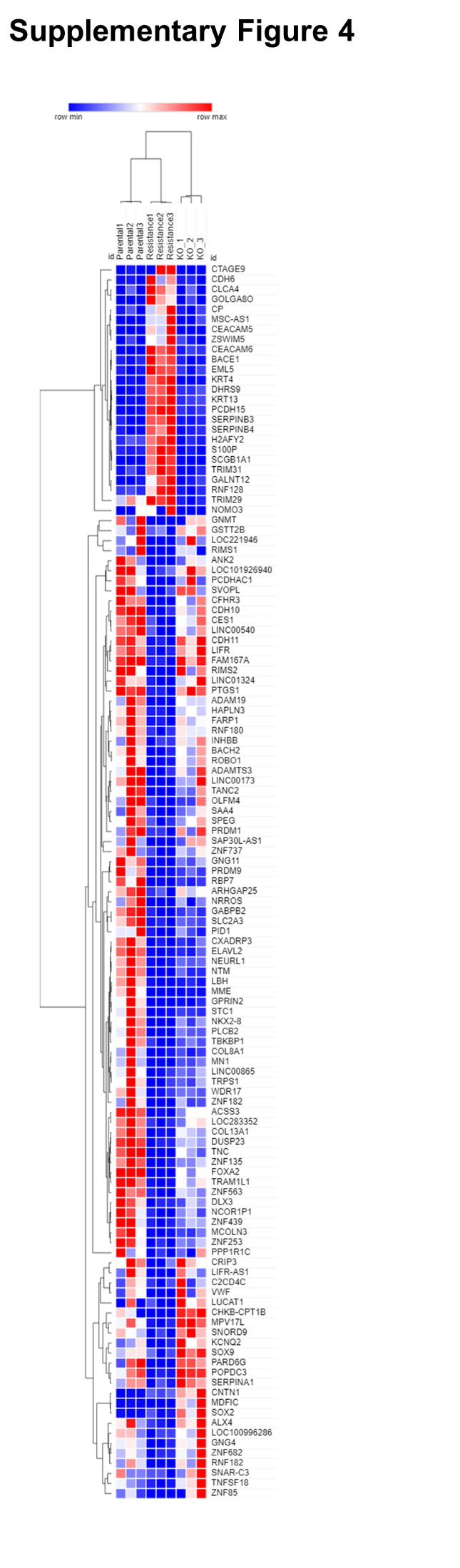
**

**Supplementary Figure 4. RNA-seq analysis shows that modulation of TRIM29 in chemoresistant TNBC cells alters multiple genes.** Heatmap showing hierarchical clustering of the DEGs specifically downregulated/upregulated in HCC1806-CarboR cells whose expression exhibits opposite trends upon TRIM29 knockout, and similar to HCC1806 cells.
