# Supplementary material for: TRIM29 upregulation contributes to chemoresistance in triple negative breast cancer via modulating S100P-β-catenin axis
Source: Cell Commun Signal. 2025 May 26;23:244. doi: 10.1186/s12964-025-02233-9 (PMC12107940; doi:10.1186/s12964-025-02233-9)
Supplement: Supplementary file 6 — Supplementary Material 6 [file 12964_2025_2233_MOESM6_ESM.docx]

**
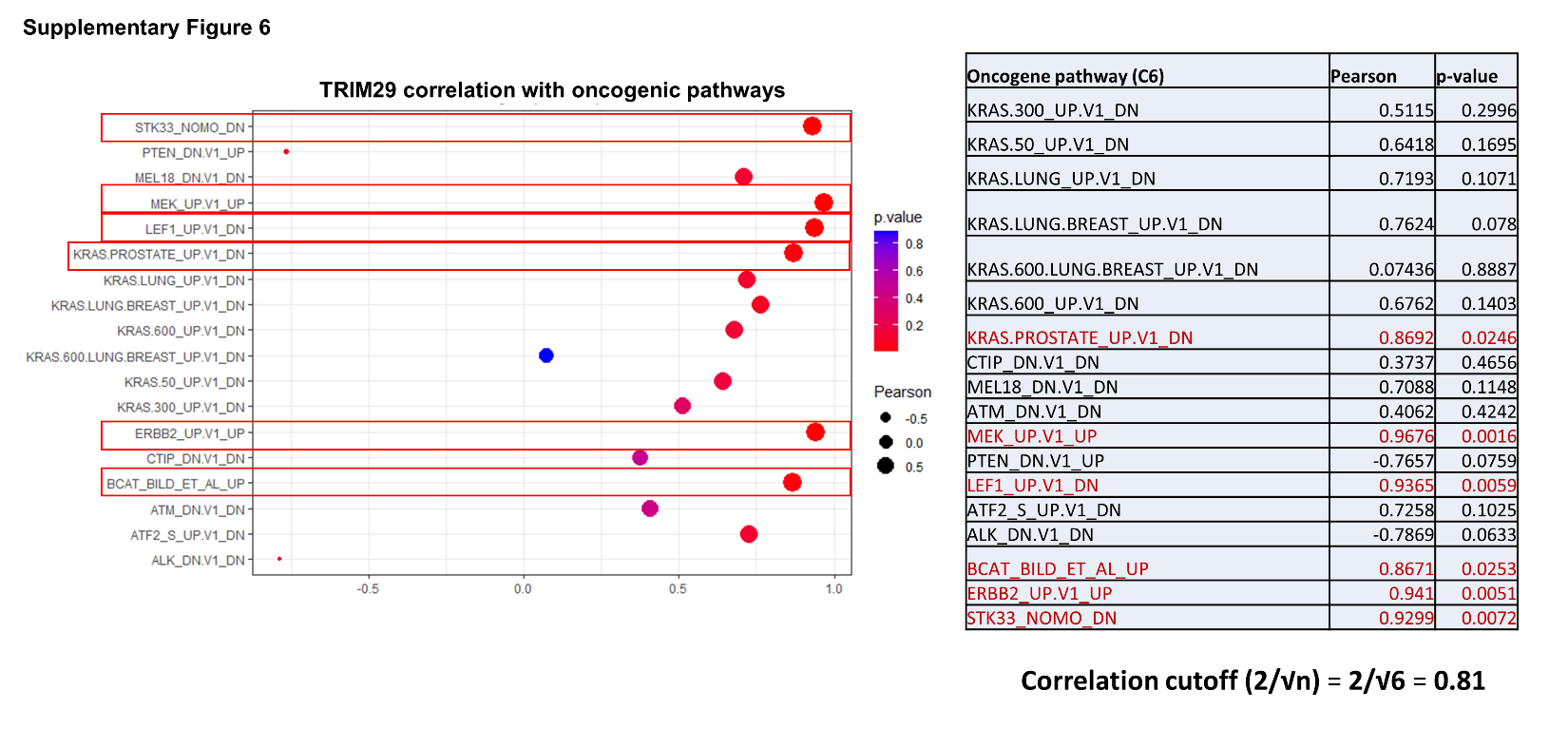
**

**Supplementary Figure 6:** Pearson correlation analysis between TRIM29 and C6 oncogenic signature gene-set using ssGSEA. The dots represent the significantly correlated oncogenic pathways with TRIM29. The highlighted pathways represents significantly correlated pathways with a correlation cutoff ≥ 0.81, p value ≥ 0.05.
