# Supplementary material for: TRIM29 upregulation contributes to chemoresistance in triple negative breast cancer via modulating S100P-β-catenin axis
Source: Cell Commun Signal. 2025 May 26;23:244. doi: 10.1186/s12964-025-02233-9 (PMC12107940; doi:10.1186/s12964-025-02233-9)
Supplement: Supplementary file 7 — Supplementary Material 7 [file 12964_2025_2233_MOESM7_ESM.docx]

**
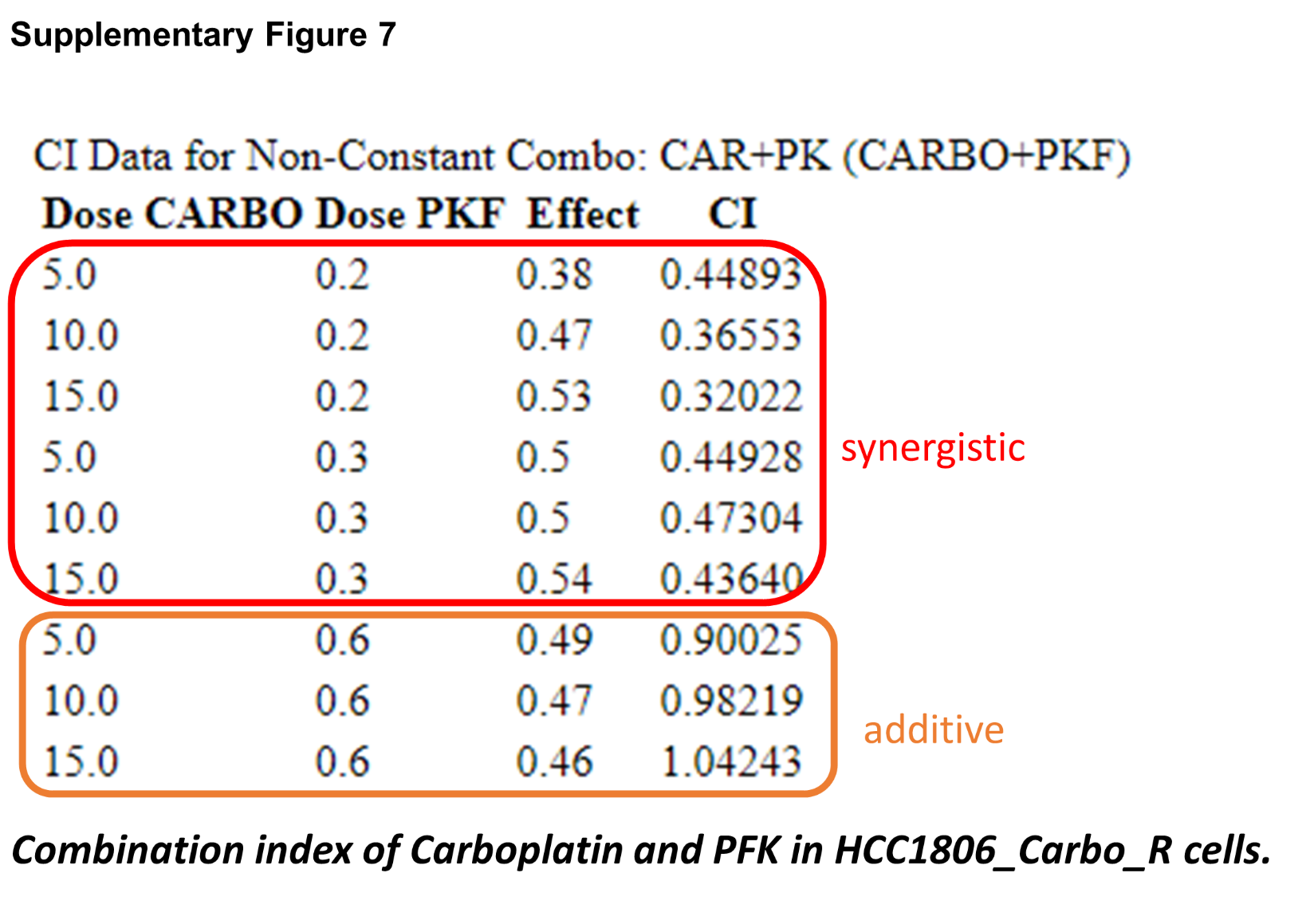
**

**Supplementary Figure 7:** HCC1806-CarboR cells were treated with a varied concentration of Carboplatin and PKF118-310, either alone or in combination. Cells were then subjected to MTT assay and the combination index (CI) was calculated using the Chou-Talalay method using CompuSyn software. CI < 1 represents synergistic effects, CI > 1 represents antagonistic effects and CI = 1 represents additive effects. Data boxed/highlighted in red represents synergistic interaction between Carboplatin and PKF118-310 while data boxed/highlighted in orange represents additive interaction between the two drugs.
