# Supplementary material for: TRIM29 upregulation contributes to chemoresistance in triple negative breast cancer via modulating S100P-β-catenin axis
Source: Cell Commun Signal. 2025 May 26;23:244. doi: 10.1186/s12964-025-02233-9 (PMC12107940; doi:10.1186/s12964-025-02233-9)
Supplement: Supplementary file 8 — Supplementary Material 8 [file 12964_2025_2233_MOESM8_ESM.docx]

**
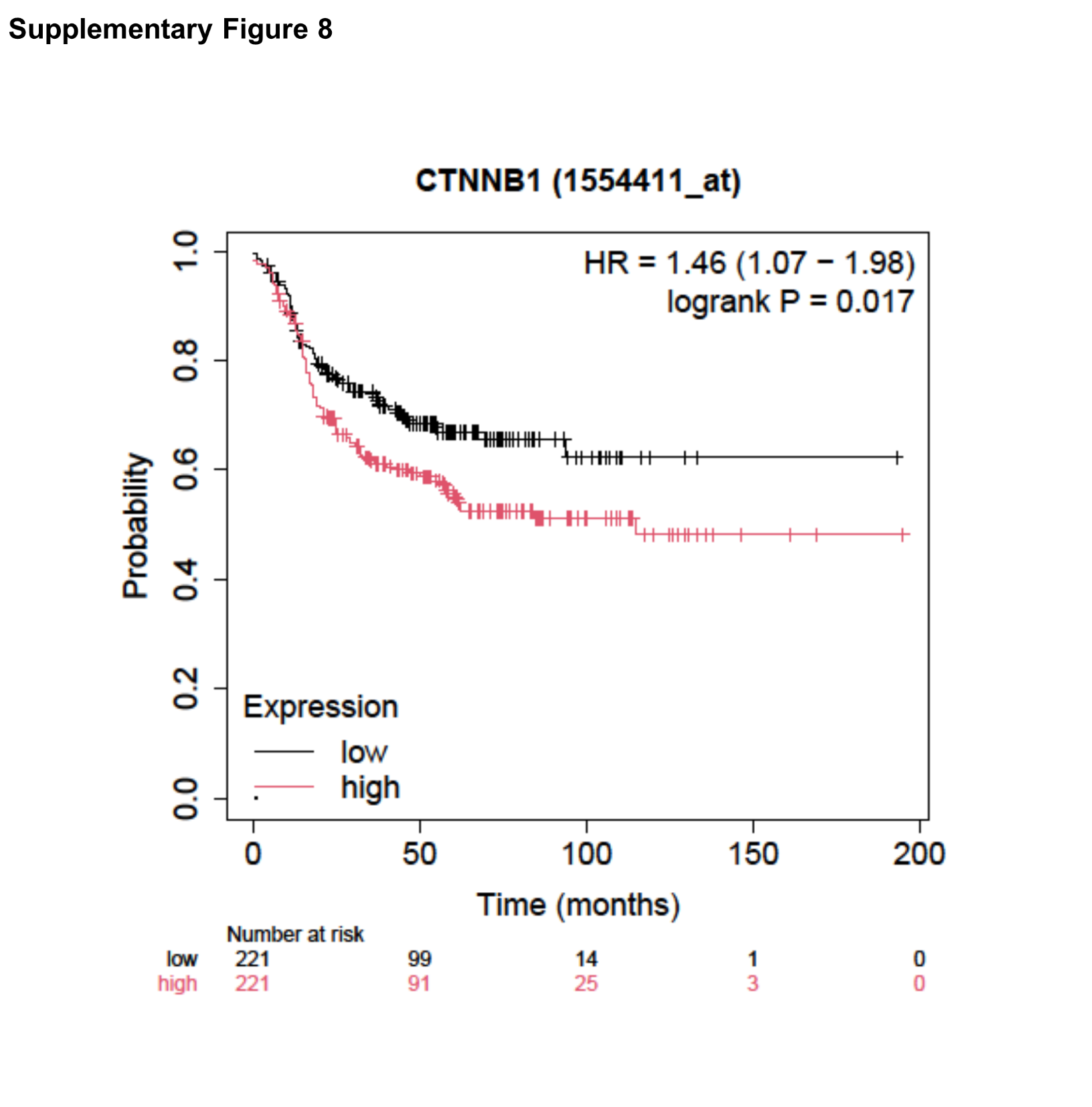
**

**Supplementary Figure 8. High expression of β-catenin correlates with poor survival.** Kaplan-Meier curves indicating TNBC (as characterized with PAM50) recurrence-free survival with high- or low- expression of β-catenin. Log-rank tests of survival patterns were used to obtain the p values.
